# Outcome measurement instruments for peripheral vascular malformations and an assessment of the measurement properties: a systematic review

**DOI:** 10.1007/s11136-019-02301-x

**Published:** 2019-09-23

**Authors:** Sophie E. R. Horbach, Amber P. M. Rongen, Roy G. Elbers, Chantal M. A. M. van der Horst, Cecilia A. C. Prinsen, Phyllis I. Spuls, Collaborators OVAMA consensus group, F. Blei, C. J. M. van der Vleuten, I. J. Frieden, G. T. Richter, S. T. Tan, T. Muir, A. J. Penington, L. M. Boon

**Affiliations:** 1 Department of Plastic, Reconstructive and Hand Surgery, Academic Medical Center, University of Amsterdam, Amsterdam, The Netherlands; 2 Department of Clinical Epidemiology, Biostatistics and Bioinformatics, Academic Medical Center, University of Amsterdam, Amsterdam, The Netherlands; 3 Department of Epidemiology and Biostatistics, Amsterdam Public Health Research Institute, VU University Medical Centre, Amsterdam, The Netherlands; 4 Department of Dermatology, Academic Medical Center, University of Amsterdam, Amsterdam, The Netherlands

**Keywords:** Vascular malformations, Outcome instruments, Outcome measurement instruments, Patient reported, PROMs, Physician reported, Measurement properties, Validity, Reliability, Responsiveness, Interpretability

## Abstract

**Purpose:**

The Outcome measures for vascular malformation (OVAMA) group reached consensus on the core outcome domains for the core outcome set (COS) for peripheral vascular malformations (venous, lymphatic and arteriovenous malformations). However, it is unclear which instruments should be used to measure these domains. Therefore, our aims were to identify all outcome measurement instruments available for vascular malformations, and to evaluate their measurement properties.

**Methods:**

With the first literature search, we identified outcomes and instruments previously used in prospective studies on vascular malformations. A second search yielded studies on measurement properties of patient- and physician-reported instruments that were either developed for vascular malformations, or used in prospective studies. If the latter instruments were not specifically validated for vascular malformations, we performed a third search for studies on measurement properties in clinically similar diseases (vascular or lymphatic diseases and benign tumors). We assessed the methodological quality of these studies following the Consensus-based Standards for the selection of health Measurement Instruments methodology, and evaluated the quality of the measurement properties.

**Results:**

The first search yielded 27 studies, none using disease-specific instruments. The second and third search included 22 development and/or validation studies, concerning six instruments. Only the Lymphatic Malformation Function Instrument was developed specifically for vascular malformations. Other instruments were generic QoL instruments developed and/or partly validated for clinically similar diseases.

**Conclusions:**

Additional research on measurement properties is needed to assess which instruments may be included in the COS. This review informs the instrument selection and/or the development of new instruments.

**Systematic review registration:**

PROSPERO, 42017056242.

**Electronic supplementary material:**

The online version of this article (10.1007/s11136-019-02301-x) contains supplementary material, which is available to authorized users.

## Introduction

Vascular malformations are benign congenital anomalies of the vascular system that are categorized based on the type of vascular channels involved: arterial, venous, lymphatic or capillary vessels, singularly or combined [1–4]. The disease impact varies, including cosmetic concerns, pain and functional impairment, depending on the size and location of the lesion. Multiple treatment options are available, including compressive stockings, sclerotherapy and surgery; however, evidence is lacking to support clinical decision-making.

In current literature, there is no uniformity regarding the outcomes that are measured to evaluate treatment effectiveness (‘outcome domains’) and what measurement methods or tools are used to measure these outcomes (also known as ‘outcome measurement instruments’ or simply‘instruments’. For this reason, outcomes of different clinical trials cannot be pooled, which hampers the development of evidence-based guidelines.

The Outcome Measures for VAscular MAlformations (OVAMA) group initiated the OVAMA project aiming to develop an international ‘Core Outcome Set’ (COS) for adult as well as pediatric patients with peripheral congenital soft tissue vascular malformations for measuring outcomes of therapeutic interventions globally. A COS is an agreed minimum set of outcomes that should be measured and reported in all clinical trials of a specific disease or trial population [5]. In an earlier e-Delphi study, the OVAMA consensus group reached consensus on the core outcome domains for patients with venous, lymphatic, and arteriovenous malformations (VM, LM, and AVM respectively): *radiological assessment*, *physician*-*assessed location*-*specific signs*, *patient*-*reported pain*, *overall severity of symptoms*, *health*-*related quality of life* (HRQoL), *patient satisfaction with treatment and outcome*, and *adverse events* (Online Resource 1). For each unique type of vascular malformation, specific physician- and patient-reported signs and symptoms were included separately. Furthermore, *recurrence* and *appearance* were recommended outcome domains based on the e-Delphi study but require further discussion before final inclusion in the COS [6]. However, it is unclear which instruments are most suitable for measuring the core outcome domains in adults and children.

This review, as part of the OVAMA project (Online Resource 2), aims to identify the outcome domains and instruments for vascular malformations that were used in previous prospective studies, and to assess the quality of the available patient- and physician-reported outcome instruments, to inform the selection process of instruments to measure the core outcome domains in future studies.

## Materials and methods

The OVAMA project was registered at the Core Outcome in Effectiveness Trials (COMET) database (http://www.comet-initiative.org/), designed following the Harmonizing Outcome Measures for Eczema (HOME) roadmap, and embedded within the Cochrane Skin Group—Core Outcome Set Initiative (CSG-COUSIN) [7]. We followed the guidelines of the PRISMA-P statement [8], the Core Outcome Set—STAndards for Reporting (COS-STAR) [9], and the COnsensus-based Standards for the selection of health Measurement INstruments (COSMIN) ‘Protocol for Systematic Reviews of Measurement Properties’ [10]. The study protocol was registered in PROSPERO (42017056242).

### Literature searches, study selection and data extraction

This systematic review consisted of three literature searches (Online Resource 3), as described below.

All searches were performed with the help of a clinical librarian. The PubMed function ‘Similar Articles’ and reference lists of all included articles were screened for additional studies. Study selection and data extraction were performed by two independent reviewers. Disagreements were resolved by consensus.

#### Search 1: identification and description of outcome domains and instruments used in previously published studies

In the first search, prospective studies evaluating treatment outcomes in vascular malformations were identified to collect all outcome domains and instruments that were previously used. We searched MEDLINE, EMBASE and CENTRAL for studies measuring treatment effectiveness, using search terms for peripheral vascular malformations. We included prospective studies evaluating treatments that enrolled at least 20 patients (all ages) with all singular and combined types of vascular malformations. Capillary, visceral, bone and central nervous system vascular malformations were excluded as these were outside the scope of the OVAMA project. Articles published before 1996 were excluded, since the current classification and terminology for vascular anomalies was established in 1996 by the International Society for the Study of Vascular Anomalies (ISSVA) [11]. Data on study characteristics, outcome domains and instruments were extracted.

#### Searches 2 and 3: evaluation of the quality of the identified outcome measurement instruments

The second search was performed in MEDLINE and EMBASE to identify development and validation studies of patient- and physician-reported disease-specific instruments for vascular malformations, using search terms for vascular malformations and a validated PubMed search filter for finding studies on measurement properties, developed by Terwee et al. [12].

The third search aimed to identify development and validation studies of patient- and physician-reported outcome measurement instruments that were used in previously published prospective studies on vascular malformations, as identified in search 1, but were not specifically developed and validated for vascular malformations. To explore their potential applicability in vascular malformations, we additionally searched for studies on measurement properties in patient populations that share clinical similarities with vascular malformations, predefined as: ‘benign vascular diseases’, ‘benign lymphatic diseases’ and ‘benign soft tissue tumors’. The expert group considered these groups of health conditions to have the greatest similarity to vascular malformations in terms of clinical appearance, signs, symptoms and potential disease burden. Search terms for the identified patient- and physician-reported instruments were combined with terms for the predefined clinically similar diseases, and the abovementioned PubMed filter for studies on measurement properties [12]. Only studies reporting on at least one measurement property of an instrument that was developed for vascular malformations, or for a clinically similar condition but previously used for vascular malformations, were included. Studies not reporting on measurement properties and studies focusing on health conditions other than vascular malformations or the predefined clinically similar conditions were excluded.

Data on characteristics of the included instruments, study samples and the study results concerning measurement properties were extracted.

### Evaluation of the methodological quality of the included studies

The methodological quality of the included studies on measurement properties was evaluated by two authors independently using the COSMIN checklist (www.cosmin.nl) [13, 14]. With this checklist, we evaluated, for each included study separately, which measurement properties were investigated (following the COSMIN taxonomy, Online Resource 4) and if the methods to do so were appropriate. Several items per measurement property were rated using on a 4-point rating scale ranging from ‘excellent’ to ‘poor’. The overall score for each measurement property was determined by the ‘worst score counts’ principle [15]. As a gold standard for patient-reported outcome measures is lacking, criterion validity was not considered. Data on interpretability and feasibility were collected if presented in the included studies. Two reviewers independently extracted data on the measurement properties from the selected articles and evaluated methodological quality of the studies. Discrepancies were discussed with a third reviewer until consensus was reached.

### Evaluation of quality of the measurement properties

Two authors independently evaluated the quality of the measurement properties by rating the results of the analyses on measurement properties in each included study based on the criteria for good measurement properties as recommended by Terwee et al. [16, 17] (Online Resource 5). The study results were independently rated by two reviewers as ‘positive’ (+), ‘negative’ (−) or ‘indeterminate’ (?) for each measurement property.

### Best evidence synthesis

The best evidence synthesis is aimed at reaching a conclusion about the overall quality of each of the measurement properties of the included instruments. For this purpose, the quality assessments of the measurement properties based on the study results of the included studies were combined and adjusted for the methodological quality of the studies by applying levels of evidence as recommended by the COSMIN group [16], taking into account the number of studies, the methodological quality of the studies and the consistency of the study results on measurement properties across studies.

For each measurement property, the methodological quality of the study (*poor, fair, good or excellent*) and the direction of the study results of the analyses on this measurement property (*negative, indeterminate or positive result*) were combined into the best evidence synthesis (Table 1): +++, ++, +: positive rating indicating “*adequate*” measurement property; ?: unknown rating indicating indeterminate measurement property; − − −, − −, −: negative rating indicating “*inadequate*” measurement property; ± : conflicting findings; NI: not interpretable (due to indeterminate result of analysis); NA: not available. (analysis was not performed for this measurement property).

**Table 1:** Levels of evidence for the overall quality of a measurement property (www.cosmin.nl) [47]

Level	Rating^a^	Criteria
Strong	+++ or − − −	Consistent findings in multiple studies of at least good quality OR one study of excellent quality
Moderate	++ or −	Consistent findings in multiple studies of fair methodological quality OR one study of good methodological quality
Limited	+ or −	One study of fair methodological quality
Conflicting	±	Conflicting findings
Unknown	?	Only studies of poor methodological quality

^a^+ positive/good, ? indeterminate, − negative/poor, ± conflicting ratings

## Results

### Search 1: identification of outcome domains and instruments

**Fig. 1:**
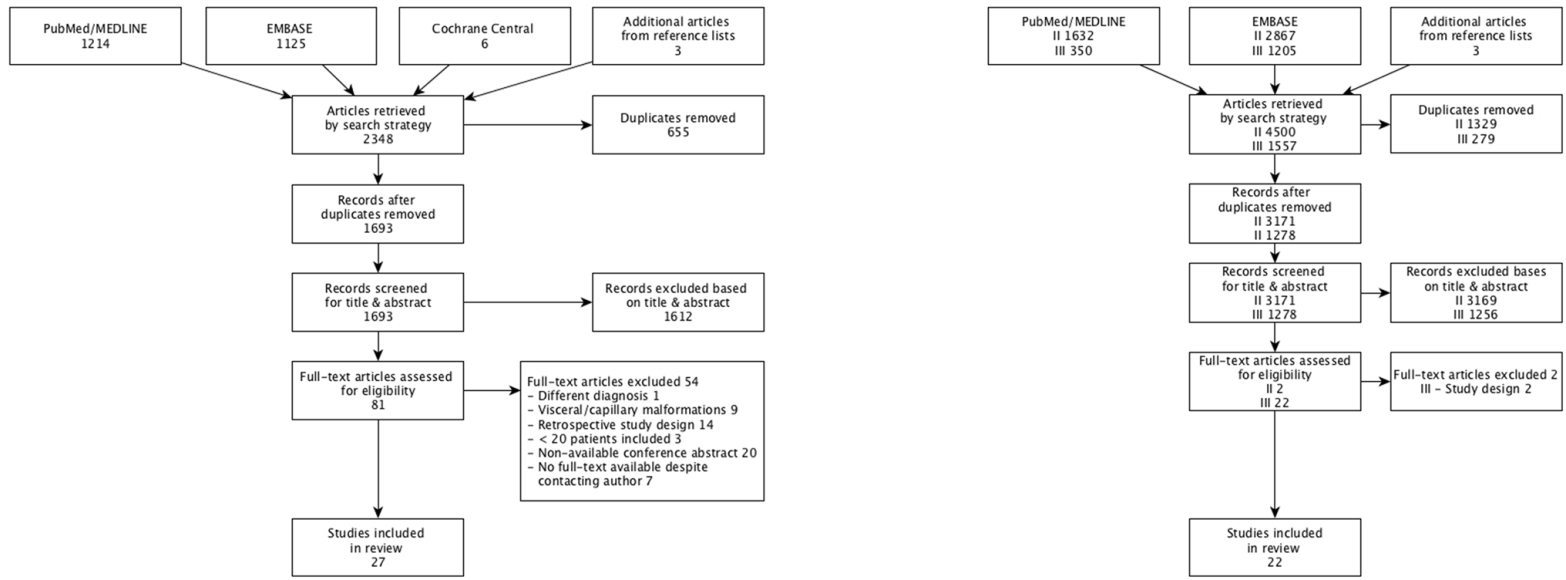
Flowcharts of study selection. Search 1 (left): the identification of instruments previously used in prospective studies on vascular malformations. Search 2 and 3 (right): the identification of development and/or validation studies of disease-specific instruments for vascular malformations (II), and development and/or validation studies for instruments previously used in prospective studies on vascular malformations (III)

In 26 of the 27 studies identified by search 1 (Fig. 1) the authors exclusively used outcomes that were recommended or selected as core outcome domains in the OVAMA e-Delphi study [6], however, inconsistently across the studies, measuring at least one of the following: *adverse events* [100% of studies], *radiological assessment* [56%], *appearance* [52%], *patient*-*reported symptoms including pain* [37%], *patient satisfaction with treatment and/or outcome* [26%], *physician*-*reported signs* [15%], *HRQoL* [15%] and *recurrence* [7%]. In one study, ‘healthcare costs’ was the primary outcome, categorized under the domain *practical issues* [4%] [18], which was not selected as a core domain in the OVAMA e-Delphy study. All instruments used for each outcome domain are listed in Table 2. None of these were disease-specific instruments for vascular malformations. Published ‘named’ instruments were only available for the assessment of HRQoL. Instruments for other outcome domains were unnamed questionnaires that were only created for singular use by the authors of the concerning study.

**Table 2:** All instruments used in prospective studies on vascular malformations, categorized per outcome domain

Domain category	Instrument	References	Type of malformation(s)	Description of instrument	Assessor
Appearance	Physical examination (unstandardised)	[48]	LM	Clinical assessment including manual measurement of the size of the lesion in 2 dimensions	Physician
	Subjective assessment by patient (unstandardised)	[49, 50]	VM	Estimation of changes in size or distortion of normal anatomical shapes	Patient
	Questionnaire	[51]	LM, AVM	To assess changes in distortion of normal anatomical shapes	Patient
	Physician assessment based on clinical photographs	[52–55]	LM, VM, AVM, CVM, CLM, LVM, Vascular malformations not specified	Estimation of changes in size, color or cosmetic outcome	Physician
	Physical examination (unstandardised)	[49, 50, 52, 56–62]	LM, VM, AVM, CLM, Vascular malformations not specified	Estimation of changes in size, normal anatomical shapes, appearance, discoloration and/or texture	Physician
HRQoL	CIVIQ-20	[41]	LM	HRQoL questionnaire for patients with chronic venous insufficiency	Patient
	EQ-5D	[18]	VM	Generic HRQoL questionnaire	Patient
	SF-36v2	[18]	VM	Generic HRQoL questionnaire	Patient
	SF-36v2	[63]	VM	Generic HRQoL questionnaire	Patient
	SF-10	[63]	VM	Parent-completed HRQoL questionnaire intended for children between ages of 5–18	Patient
	PedsQL4.0	[64]	LM, CLVM, LVM	HRQoL questionnaire for children aged 2–18 years	Patient
	PedsQL4.0 Infant Scales	[64]	LM, CLVM, LVM	HRQoL questionnaire for children ages 1–12 months and 13–24 months	Patient
	FACIT	[64]	LM, CLVM, LVM	Collection of HRQoL questionnaires targeted to the management of chronic illness	Patient
	FACT-G	[64]	LM, CLVM, LVM	Generic HRQoL questionnaire	Patient
Patient-reported symptoms	VAS	[65–67]	LM, VM	100-mm scale to assess pain and/or swelling	Patient
	Unnamed questionnaire^a^	[41, 50, 51, 56, 59, 60, 65, 68]	LM, VM, AVM	To assess changes in pain, swelling, overall severity of symptoms, functional impairment, visual-, breathing-, swallowing- or speaking problems and/or limb function	Patient
Patient satisfaction with treatment and outcome	Unnamed questionnaire^a^	[41, 49, 60, 65, 66]	LM, VM	To assess overall benefit from treatment, satisfaction with cosmetic outcome, global result and/or acceptance^b^	Patient
	VAS	[67]	VM	100-mm scale to assess overall cosmetic outcome^b^	Patient
	Satisfaction expressed by patient	[56]	VM, AVM	Satisfaction expressed by the patient	Patient
Physician-reported signs	Physical examination (unstandardised)	[50, 51, 56, 68]	LM, VM, AVM	To assess changes in swelling, range of movement and/or unspecified clinical findings	Physician
Practical issues	Accounting system	[18]	VM	To assess medical costs of sclerotherapy procedure, hospitalization and follow-up imaging	
	Incremental cost-effectiveness ratio	[18]	VM	To calculate increment in medical cost/QALY gained	
	Utility	[18]	VM	To calculate utility from EQ-5D and time	
Radiological assessment	(Color Doppler/Duplex) USG	[48, 50, 55, 57, 58, 61, 68, 69]	LM, VM, AVM	To assess changes in lesion volume or blood flow velocity	Physician/Radiologist
	CT/MRI	[41, 49, 51, 61, 64, 67, 68, 70, 71]	LM, VM, AVM, CLVM, LVM	To assess changes in lesion volume, clinical phleboliths and/or organ compression, bone or joint deformities	Physician/Radiologist
Recurrence	Clinical assessment (unstandardised)	[50, 68]	VM, AVM	Not reported	Physician

^a^Measurement instrument which was vaguely or not specified

^b^By the authors categorized as ‘satisfaction’, possibly also classifiable under the domain ‘Appearance’ or under ‘Supplementary outcome domains’; *LM* lymphatic malformation, *VM* venous malformation, *AVM* arteriovenous malformation, *CVM* capillary-venous malformation, *CLM* capillary-lymphatic malformation, *LVM* lymphatic venous malformation, *CIVIQ*-*20* 20-item chronic venous insufficiency quality of life questionnaire, *HRQoL* health-related quality of life, *EQ*-*5D* EuroQuality of life 5-dimensions, *VAS* visual analog scale, *SF*-*36v2* Short-Form 36 health survey version 2, *SF*-*10* Short-Form 10 health survey for children, *CLVM* capillary lymphatic venous malformation, *PedsQL4.0* pediatric quality of life inventory 4.0, *FACIT* functional assessment of chronic illness therapy; *FACT*-*G* functional assessment of cancer therapy general

### Searches 2 and 3: evaluation of the quality of the identified instruments

The searches for development and validation studies on instruments used for vascular malformations provided 4446 articles (vascular malformations *n* = 3170; similar diseases *n* = 1276), the major reasons for exclusion were failure to report on measurement properties and studies investigating unrelated health conditions. Twenty-two studies were included [19–40] evaluating six different instruments (Fig. 1), of which there was only one disease-specific instrument for vascular malformations. The other included instruments were four generic instruments developed or partly validated for other clinically similar diseases (two including a disease-specific module) and one disease-specific instrument for varicose veins, all previously used in vascular malformation studies. Characteristics of the selected instruments and study populations are presented in Tables 3 and 4, respectively. The methodological quality of the studies, the quality of the measurement properties and the best evidence synthesis are presented in Table 5. Details on the COSMIN ratings and the evaluation of the measurement properties per included study can be found in Online Resource 6.

**Table 3:** Characteristics of the identified ‘named’ patient- and physician-reported outcome measurement instruments that were developed or previously used for vascular malformations, for which studies on measurement properties were available

Instrument	Target population	Domains	No. of items	Scoring method	Total score range	Recall period (days)
LMF [27, 40]	Cervicofacial lymphatic malformations	Functional impairment (signs and impacts)	12	3-point Likert scale, ordinal	0–2, with 0 = best health and 2 = worst health	30
CIVIQ-20 [22, 24–26, 29, 30, 37, 39]	CVI	Physical, psychological and social impairment, pain	20	5-point Likert scale, ordinal	0–100, with 100 = worst QoL and 0 = best QoL	28
SF-36 [20, 21, 28, 32–36, 38]	Clinical practice and research, health policy evaluations and general population surveys	PF, PA, BP, GH, V, SF, EWB, MH	36	NR	0–100, with 0 = worst QoL and 100 = best QoL	NR
EQ-5D [28, 31, 35]	NR	Mobility, self-care, usual activities, pain, anxiety	5	3-point Likert scale, ordinal	Responses fitted into an equation developed from a regression model which produces a score from − 0.59 to 1.00, with − 0.59 = worst QoL, 1.00 = best QoL and 0 = death	NR
PedsQL-NF1, adults [23]	Neurofibromatosis type 1	Physical, emotional, social, cognitive functioning, communication, worry, perceived physical appearance, pain and hurt, paresthesia’s, skin irritation, sensation, movement and balance, daily activities, fatigue, treatment anxiety, sexual functioning	74	5-point likert scale, ordinal	0–100, with 0 = worst QoL and 100 = best QoL	NR
PedsQL-NF1, children, adolescents and young adults [19]	Neurofibromatosis type 1	Skin, pain, pain impact, pain management, cognitive functioning, speech, fine motor, balance, vision, perceived physical appearance, communication, worry, treatment, medicines, gastrointestinal symptoms	115	3-point likert scale, ordinal	UD	UD

*LMF* lymphatic malformation function instrument, *CIVIQ*-*20* 20-item chronic venous insufficiency quality of life questionnaire, *CVI* chronic venous insufficiency, *QoL* quality of life, *SF*-*36* Short-Form 36 health survey, *PF* physical functioning, *PA* physical aspects, *BP* bodily pain, *GH* general health, *V* vitality, *SF* social functioning, *EWB* emotional well-being, *MH* mental health, *NR* not reported, *EQ*-*5D* EuroQuality of life 5-dimensions, *PedsQL*-*NF1* pediatric quality of life neurofibromatosis type 1, *UD* under development

**Table 4:** Characteristics of the test populations of the included studies

Author	Country	Language version	*n*	Percentage of vascular malformations or similar diseases (%)	Mean age (years)	Gender (% male)	Disease characteristics	Setting
The Lymphatic Malformation Function Instrument (LMF)								
Balakrishnan K, 2012 [40]	USA	English	35	100	9	NR	Cervicofacial lymphatic malformations	Pediatric hospital vascular anomalies clinic
Kirkham EM, 2015 [27]	USA	English	60	100	7	50	Cervicofacial lymphatic malformation, without previously complete surgical resection	Tertiary care centers and online
The 20-item chronic venous insufficiency quality of life questionnaire (CIVIQ-20)								
Launois R, 1996 (1) [25]	France	French	1001	100	NR	NR	Symptomatic CVI	NR
Launois R, 1996 (2) [25]	France	French	934	100	NR	NR	CVI with symptoms of heavy legs, cramping or pain or moderate functional incapacity	NR
Jantet G, 2000 (Subset Jantet, 2002) [30]	Czech and Slovak republics, Hungary, Poland, Russia and Spain	Czech, Slovak, Hungarian, Polish, Russian and Spanish	3075	100	45.7	15.5	CVI (2 groups: with and without venous reflux)	NR
Jantet G, 2002 [29]	23 countries from the European, African, American and Asian continents		5052	100	45.2	20	CVI	NR
Lozano FS, 2002 (Subset Jantet G, 2002) [24]	Spain	Spanish	476	100	NR	NR	CVI	NR
Erevnidou K, 2004 [37]	Greece	Greek	50	100	61.1	14	CVI	2 vascular surgery clinics
Launois R, 2010 (Subset Jantet, 2002) [26]	18 countries^a^		4048	100	45.6	18.9	CVI	Surgical outpatient clinic
Ozdemir OC, 2016 [22]	Turkey	Turkish	140	100	52.3	27.9	Patients ≥ 18 years old with CVI CEAP C stages C3–C6	Hospital
Biemans AAM, 2011 [39]	The Netherlands	Dutch	159	100	53	29.60%	New patients who consulted departments of dermatology and vascular surgery for varicose veins	Hospital
The Short-Form 36 (SF-36) health survey								
Garratt AM, 1993[34]	United Kingdom	English	1746	21	42.7	33.5	Patients suffering from low back pain, menorrhagia, suspected peptic ulcer or varicose veins	Referral letters and general practitioners
Garratt AM, 1994[33]	United Kingdom	English	1148	20	47.9	46.1	Patients suffering from low back pain, menorrhagia, suspected peptic ulcer or varicose veins	Referral letters and general practitioners
Ruta DA, 1994[21]	United Kingdom	English	1746	21	42.7	33.9	Patients suffering from low back pain, menorrhagia, suspected peptic ulcer or varicose veins	Referral letters and general practitioners
Garratt AM, 1996[32]	USA	English	368	100	45.8	24	Varicose veins	Outpatient clinic and general practitioner
Walters SJ, 1999[20]	United Kingdom	English	233	100	75 (median)	33.5	Venous leg ulcers	8 community clinics
Jull A, 2010 (2) [28]	New Zealand	English	368	100	68	49%	Venous leg ulcers	Community-based district nursing services
Franks PJ, 2003 [36]	United Kingdom	English	118	100	78	26.3	Patients suffering from chronic leg ulceration	Hospital and community services
Franks PJ, 2006 (1) [35]	United Kingdom	English	189	100	76.7 (median)	29.3%	Lymphedema of the lower limb	Hospital and outpatient clinic
Qiang cao, 2013 [38]	China	Chinese	179	100	58	NR	Patients first suffering from deep venous thrombosis	Hospital
Euro quality of life 5 domains (EQ-5D)								
Iglesias CP, 2005 [31]	United Kingdom	English	387	100	71.6	41	Chronic venous leg ulcers	NR
Jull A, 2010 (1) [28]	New Zealand	English	368	100	68	49%	Venous leg ulcers	Community-based district nursing services
Franks PJ, 2006 (2) [35]	United Kingdom	English	189	100	76.7 (median)	29.3%	Lymphedema of the lower limb	Hospital and outpatient clinic
Pediatric quality of life inventory neurofibromatosis type 1 (PedsQL NF1, adults)								
Nutakki K, 2013 (1)[23]	USA	English	10	100	40.5	NR	Adults with neurofibromatosis type 1	Children’s Tumor Foundation sponsored neurofibromatosis forum
Nutakki K, 2013 (2)[23]	USA	English	11	100	40.2	NR	Adults with neurofibromatosis type 1	Hospital, conferences, online
Pediatric quality of life inventory neurofibromatosis type 1 (PedsQL NF1, children, adolescents and young adults)								
Nutakki K, 2017 [19]	USA	English	90	100	NR	NR	Patients with neurofibromatosis type 1 between 5 and 25 years and parents of children ages 5–17 years	Hospital

*NR* not reported, *CVI* chronic venous insufficiency, *CEAP* comprehensive classification system for chronic venous disorders

^a^Argentina, Brazil, Brunei, Czech Republic, Egypt, Hong Kong, Hungary, India, Malaysia, Philippines, Poland, Russia, Singapore, Slovakia, Spain, Sri Lanka, Turkey and Venezuela

**Table 5:** Methodological quality, quality assessment of measurement properties and best evidence synthesis per instrument and per disease

Author, year	Internal consistency	Reliability	Measurement error	Content validity	Structural validity	Hypotheses testing	Cross-cultural validity	Responsiveness
The lymphatic malformation function instrument (LMF)								
Cervicofacial lymphatic malformations								
Balakrishnan K, 2012 [40]				Excellent (+)				
Kirkham EM, 2015 [27]	Fair (+)	Poor (+)			Fair (−)	Fair (−)		
Best evidence synthesis	+	?	NA	+++	−	−	NA	NA
The 20-item chronic venous insufficiency quality of life questionnaire (CIVIQ-20)								
Chronic venous insufficiency								
Launois R, 1996 (1) [25]	Fair (+)	Fair (?)			Fair (+)	Poor (+)		
Launois R, 1996 (2) [25]	Fair (+)			Good (+)	Fair (+)			poor (?)
Jantet G, 2000 [30]	Fair (+)	Fair (+)			Fair (?)	Poor (+)	Poor (+)	Fair (?)
Jantet G, 2002 [29]	Poor (+)	Fair (+)			Poor (?)	Fair (+)		Fair (+)
Lozano FS, 2002 [24]	Fair (+)	Fair (+)					Poor (?)	Poor (?)
Erevnidou K, 2004 [37]	Fair (+)	Fair (?)			Poor (−)	Fair (+)	Poor (?)	
Launois R, 2010 [26]	Good (+)	Good (+)			Good (?)	Good (+)		Fair (+)
Ozdemir OC, 2016 [22]	Poor (+)	Fair (+)	Fair (?)		Fair (+)	Fair (+)	Fair (+)	
Best evidence synthesis	++	++	NI	++	++	++	+	++
Varicose veins								
Biemans AAM, 2011 [39]	Poor (+)	Good (?)			Good (?)	Fair (+)	Good (−)	Fair (+)
Best evidence synthesis	?	NI	NA	NA	NI	+	− −	+
The Short-Form 36 (SF-36) health survey								
Varicose veins								
Garratt AM, 1993 [34]	fair (+)				Fair (?)	Fair (+)		
Garratt AM, 1994 [33]								Fair (+)
Ruta DA, 1994 [21]	poor (+)	fair (?)						
Garratt AM, 1996 [32]		fair (+)				Fair (+)		Fair (+)
Best evidence synthesis	+	+	NA	NA	NI	++	NA	++
Venous leg ulcers								
Walters SJ, 1999 [20]	poor (+)					Good (+)		Good (−)
Jull A, 2010 (2) [28]								Fair (+)
Franks PJ, 2003 [36]	poor (+)							Fair (?)
Best evidence synthesis	?	NA	NA	NA	NA	++	NA	±
Lymphedema of the lower limb								
Franks PJ, 2006 (1) [35]	Poor (+)					Poor (+)		Fair (+)
Best evidence synthesis	?					?		+
Deep venous thrombosis								
Qiang cao, 2013 [38]	Excellent (+)	Good (+)			Good (?)			Poor (?)
Best evidence synthesis	+++	++	NA	NA	NI	NA	NA	NI
The euro quality of life—5 domain (EQ-5D)								
Venous leg ulcers								
Iglesias CP, 2005 [31]						Fair (−)		Fair (+)
Jull A, 2010 (1) [28]								Fair (+)
Best evidence synthesis	NA	NA	NA	NA	NA	–	NA	++
Lymphedema of the lower limb								
Franks PJ, 2006 (2) [35]						Fair (+)		Fair (+)
Best evidence synthesis	NA	NA	NA	NA	NA	+	NA	+
Pediatric quality of life inventory neurofibromatosis type 1 (PedsQL NF1, adults)								
Neurofibromatosis type 1								
Nutakki K, 2013 (1) [23]	Poor (+)			Good (+)				
Nutakki K, 2013 (2) [23]	Excellent (+)				Excellent (?)	Fair (+)		
Best evidence synthesis	+++	NA	NA	++	?	+	NA	NA
Pediatric quality of life inventory neurofibromatosis type 1 (PedsQL NF1, children, adolescents and young adults)								
Neurofibromatosis type 1								
Nutakki K, 2017 [19]				Excellent (+)				
Best evidence synthesis	NA	NA	NA	+++	NA	NA	NA	NA

For each measurement property, the methodological quality of the study is reported as ‘poor’, ‘fair’, ‘good’ or ‘excellent’. The ratings of the study results of the analyses on this measurement property are depicted between brackets as ‘−‘(negative result),’?’(indeterminate result), ‘+’(positive result). These two ratings were combined into the best evidence synthesis: +++, ++, + positive rating indicating adequate measurement property; ? unknown rating indicating indeterminate measurement property; − − −, − −, − negative rating indicating inadequate measurement property; ±  conflicting findings, *NI* not interpretable (due to indeterminate result of analysis), *NA* not available. (analysis was not performed for this measurement property)

#### Lymphatic malformation function instrument (LMF)

The LMF is a disease-specific questionnaire to assess functional and clinical signs and subsequent impact on daily life in pediatric patients with cervicofacial LMs. We found strong evidence for adequate content validity [40]. Because it was not described how missing values were handled, there was limited evidence for adequate internal consistency, for inadequate structural validity, and for inadequate hypotheses testing. The evidence for test–retest reliability was unknown, as the analysis was only performed in seventeen patients and therefore the methodological quality of the study on reliability was poor [27]. Data on interpretability, responsiveness to changes over time or feasibility were lacking.

#### 20-item chronic venous insufficiency QoL questionnaire (CIVIQ-20)

The CIVIQ-20 is a disease-specific HRQoL questionnaire for patients with chronic venous insufficiency (CVI), which was validated in patients with CVI [22, 24–26, 29, 30, 37] and in (isolated) varicose veins [39]. In CVI, moderate evidence was found for adequate internal consistency and reliability. The evidence for measurement error was rated ‘indeterminate’, as the minimal important change was not defined [22], although this is crucial for concluding if changes in score can be attributed to true changes in the construct. For chronic venous insufficiency, there was moderate evidence for adequate content validity and structural validity.

The evidence for adequate hypotheses-testing and responsiveness was rated as moderate (CVI) and limited (varicose veins), as the statistical methods used were suboptimal. Ceiling effects were found in three items, which may limit the instrument’s ability to detect changes in health. The number of missing items was low (0–9.4%).

#### Short-Form-36 health survey version 2 (SF36v2)

The SF-36 is a generic HRQoL questionnaire [20, 21, 28, 32–36, 38]. Evidence for adequate internal consistency varied from unknown (venous leg ulcers and lymphedema of the lower limb) [20, 35, 36], limited (varicose veins) [21, 34] to strong (deep venous thrombosis (DVT)) [38]. The evidence for adequate reliability was considered limited for varicose veins and moderate for DVT, as information on missing items was lacking. The evidence for structural validity could not be interpreted due to the lack of information about explained variance [34, 38]. Finally, limited to moderate evidence for adequate responsiveness was found in lymphedema of the lower limb [35] and varicose veins [32, 33], respectively. There was conflicting evidence for venous leg ulcers [20, 28, 36], and in DVT the evidence was not interpretable since predefined hypotheses about the expected results were lacking [38]. In the studies describing interpretability [20, 35], floor- and ceiling effects were found in three of eight dimensions. Feasibility aspects were not reported.

#### EuroQoL-5-dimensions (EQ-5D)

The EQ-5D is a generic HRQoL questionnaire, for which only 3 validation studies were performed in our target populations. In venous leg ulcers, limited evidence was available for inadequate hypotheses-testing for construct validity as no predefined hypotheses were stated for the expected correlations [31], whereas in lymphedema of the lower limb, limited evidence was found for adequate hypothesis-testing validity [35]. The evidence for adequate responsiveness was moderate and limited quality in venous leg ulcers and lymphedema of the lower limb, respectively, because the statistical methods applied were suboptimal or not appropriate [28, 31, 35]. No floor- and ceiling effects were found [35]. Feasibility aspects were not reported.

#### Pediatric QoL inventory (PedsQL) -NF1, adults

The PedsQL-NF1 is a disease-specific HRQoL questionnaire for patients with neurofibromatosis type 1 [23]. There was strong evidence for adequate internal consistency and moderate evidence for adequate content validity. Evidence for structural validity is unknown because no explained variance was presented in the included study [23]. Evidence for adequate hypotheses-testing validity was rated limited, as hypotheses were formulated in retrospect and not predefined. Feasibility, measured by the percentage of missing responses was 4.8% for all subscales [23]. Interpretability was not assessed.

#### PedsQL-NF1, children, adolescents and young adults

Finally, strong evidence for adequate content validity was found for the PedsQL-NF1 in children, adolescents and young adults [19, 23]. Information on other measurement properties is lacking.

## Discussion

All eight outcome domains which were agreed on as the core outcome domains in the OVAMA e-Delphi study were assessed in previous prospective studies. However, the outcome domains are not measured consistently throughout studies, and the instruments used differ markedly as well.

The LMF is the only partially validated disease-specific instrument available that has been developed as a composite instrument to assess signs and subsequent impact on daily activity of living in pediatric patients with cervicofacial LMs. We found strong evidence for adequate content validity in patients with cervicofacial lymphatic malformations, which is promising. Yet, future studies in larger samples should further investigate the internal consistency, reliability, measurement error, structural validity, hypotheses-testing for construct validity, cross-cultural validity and responsiveness of this instrument. The LMF was only published recently and has not yet been used in prospective studies [27]. Responsiveness has not been investigated, which is a crucial aspect when evaluating treatment outcome. A disadvantage is the applicability to a subset of pediatric patients with cervicofacial LMs only. To broaden its applicability, further validation of this instrument in other types of cervicofacial vascular malformations would be necessary.

The other included validation studies only concerned HRQoL instruments that were either generic or developed for other conditions with clinical similarities to vascular malformations. Since these instruments were not validated for vascular malformations, we evaluated the measurement properties of these instruments in similar clinical populations. The generalizability of these results may be debatable, but it reflects the best available evidence for exploring which instruments show the greatest promise for use in vascular malformation research.

For assessing HRQoL, the SF-36 is a promising measure for adult patients as its measurement properties are well-investigated in diseases that are clinically similar to vascular malformations. Fewer validation studies in smaller patient populations with clinical similar diseases were available for the EQ-5D. The SF-10, FACT-G and FACIT were previously used for vascular malformations, but have not been validated for this condition or a similar disease. They may be equally applicable to vascular malformations in terms of item relevance, comprehensiveness and comprehensibility, but to date there is no evidence to support this. For children with vascular malformations, the PedsQL was the only HRQoL instrument used. This instrument was investigated in children and parents with neurofibromatosis and seems favorable with regard to the measurement properties analyzed so far [19, 23].

The CIVIQ-20 [41] has adequate measurement properties for patients with venous insufficiency and therefore it may be worthwhile to further investigate this instrument for vascular malformations of the lower extremities. This may also apply to other instruments for similar diseases that have not yet been used for vascular malformations, like VEINES-QoL [42] or the Nottingham Health Profile [43] for varicose veins. However, the face and content validity of these instruments may be suboptimal for capturing the most important aspects for patients with vascular malformations, or may only be applicable to a specific type or location of the vascular malformation.

Interestingly, all included validation studies used classical test theory (CTT) methods, as opposed to item response theory (IRT). In CTT, measurement properties are assessed on instrument-level, depending on the items and study sample used, whereas IRT has an item-level focus [44]. The individual validation of items in IRB-based instruments facilitates computer-adaptive testing, in which the items used in the questioning process adapt to the respondent’s previous answers [45], and linkage with existing item banks such as the ‘Patient-Reported Outcomes Measurement Information System’ [PROMIS] [46].

There were no validation studies available for instruments measuring the patient- or physician-reported core domains for vascular malformations *pain*, *overall severity of symptoms*, *patient satisfaction with treatment and outcome* nor for the recommended domains *appearance* and *recurrence*. *Radiologic assessment* of the vascular malformation fell outside the scope of this study as we focused on patient/physician-subjective instruments. Further research is required to determine which radiologic imaging modalities are suitable for measuring treatment outcome.

It seems necessary to develop a new disease-specific instrument for vascular malformations, or a disease-specific attribution module that can be used alongside a generic instrument, to adequately cover all previously established core domains. In general, disease-specific instruments are also more likely to pick up small differences in quality of life caused by disease burden than the broad generic instruments.

## Conclusion

This study provides information on the available evidence for the quality of patient- and physician-reported outcome measurement instruments that have been developed or have previously been used for peripheral vascular malformations. The LMF is the only available disease-specific instrument for assessing signs and life impact in children with cervicofacial LMs. The identified generic HRQoL instruments, of which SF-36 (for adults) and PedsQL (for children) seem the most widely applicable, most investigated and promising in terms of measurement properties, may be used but it remains unclear if these instruments are responsive to treatment-induced changes in health in patients with vascular malformations. Further research into measurement properties may therefore be necessary to assess if the instruments that were identified in this systematic review are suitable for inclusion in the COS. It is likely that new disease-specific instruments need to be developed to adequately cover all core domains for vascular malformations.

The results of this review will be used as input for the future consensus meeting with all stakeholders aiming to reach consensus on the core outcome measurement instruments.

## Electronic supplementary material

Below is the link to the electronic supplementary material.

Supplementary material 1 (DOC 40 kb)

Supplementary material 2 Online Resource 2: The OVAMA project steps to develop a core set of outcome measurement instruments. COS: core outcome set; LM: lymphatic malformation; VM: venous malformation; AVM: arteriovenous malformation. (JPEG 1171 kb)

Supplementary material 3 (DOC 54 kb)

Supplementary material 4 (DOCX 15 kb)

Supplementary material 5 (DOCX 20 kb)

Supplementary material 6 (DOCX 109 kb)
