# Supplementary material for: Outcome measurement instruments for peripheral vascular malformations and an assessment of the measurement properties: a systematic review
Source: Qual Life Res. 2019 Sep 23;29(1):1–17. doi: 10.1007/s11136-019-02301-x (PMC6962285; doi:10.1007/s11136-019-02301-x)
Supplement: Supplementary file 1 — Supplementary material 1 (DOC 40 kb) [file 11136_2019_2301_MOESM1_ESM.doc]

| ***Online Resource 1.*** Core outcome domains for vascular malformations | | | | | |
| --- | --- | --- | --- | --- | --- |
| **Domain category** | **Core outcome domains included in COS** | | | | |
|  | **For all vascular malformation types** | **Specific for LM** | | **Specific for VM** | **Specific for AVM** |
| **Anatomy of the vascular malformation** | - Radiological assessment (size, flow characteristics etc.) |  | |  |  |
| **Physician-reported signs** | - Location-specific signs | - Infections - Lymphatic fluid leakage | | - Localized thrombosis | - Bleeding - Cardio-vascular health issues |
| **Patient or parent-reported symptoms** | - Pain - Overall severity of symptoms |  | |  | - Bleeding |
| **Quality of Life** | - Overall health-related QoL, including (sub)domains:   - Work  - Activities of Daily Living  - Mobility  - Emotional well-being  - Confidence |  | |  |  |
| **Satisfaction** | - Patient satisfaction with treatment - Patient satisfaction with outcome |  | |  |  |
| **Adverse events** | - All |  | | - Venous thrombo-embolism | - Mortality - Amputation |
|  | **Outcome domains recommended but requiring further discussion*** | | | | |
| **Recurrence** | - Recurrence in general |  | *Needs further specification; may be left out if it overlaps with other included domains* | | |
| **Appearance** | - Appearance as assessed by the physician - Appearance as assessed by the patient or parent (so far only consensus for LMs) |  | *Needs further discussion; contrasting results of e-Delphi surveys and online consensus meeting* | | |

List of proposed core outcome domains in e-Delphi study (Horbach et al., British Journal of Dermatology 2017). *Requires further discussion during the ISSVA conference 2018 in Amsterdam, the Netherlands.
