# Supplementary figures and images for: Outcome measurement instruments for peripheral vascular malformations and an assessment of the measurement properties: a systematic review
Source: Qual Life Res. 2019 Sep 23;29(1):1–17. doi: 10.1007/s11136-019-02301-x (PMC6962285; doi:10.1007/s11136-019-02301-x)

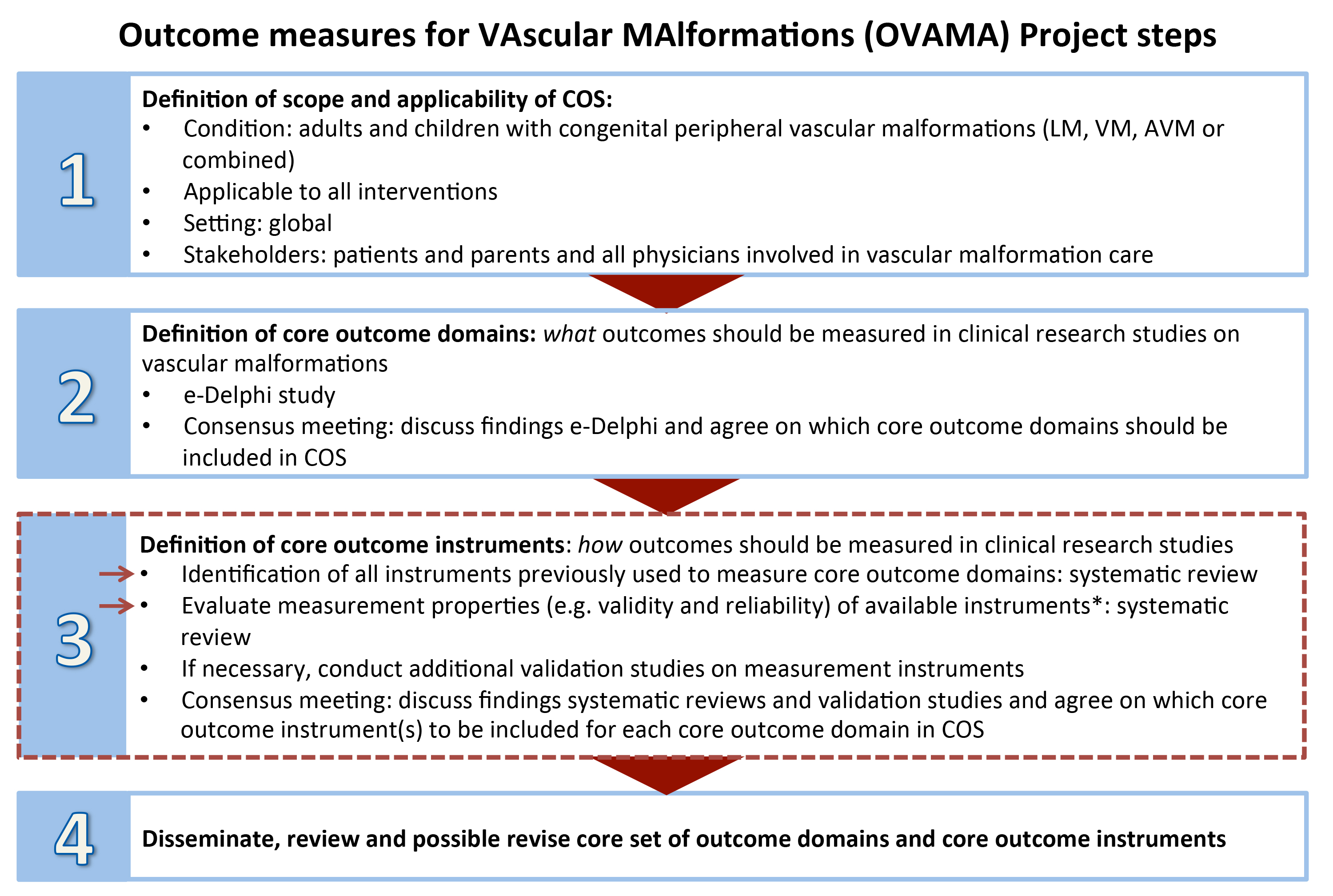

Supplement: Supplementary file 2 — Supplementary material 2 Online Resource 2: The OVAMA project steps to develop a core set of outcome measurement instruments. COS: core outcome set; LM: lymphatic malformation; VM: venous malformation; AVM: arteriovenous malformation. (JPEG 1171 kb) [file 11136_2019_2301_MOESM2_ESM.jpg]
