# Supplementary material for: Outcome measurement instruments for peripheral vascular malformations and an assessment of the measurement properties: a systematic review
Source: Qual Life Res. 2019 Sep 23;29(1):1–17. doi: 10.1007/s11136-019-02301-x (PMC6962285; doi:10.1007/s11136-019-02301-x)
Supplement: Supplementary file 3 — Supplementary material 3 (DOC 54 kb) [file 11136_2019_2301_MOESM3_ESM.doc]

**Online resource 3.** Search strategies in PubMed and EMBASE.

**Search string Search I**

**MEDLINE (14-12-2016)**

("Vascular Malformations"[Mesh] OR vascular malformation* [tiab] OR vascular anomal* [tiab] OR arteriovenous malformation* [tiab] OR vascular malformations[tiab] OR “Lymphatic Abnormalities”[Mesh] OR Lymphatic abnormal*[tiab] OR lymphatic malformation*[tiab] OR “Lymphangioma”[Mesh] OR lymphangioma*[tiab] OR venous malformation*[tiab] OR venous angioma[tiab] OR venolymphatic malformation* [tiab] NOT (cerebel*[tiab] OR cerebr* [tiab] OR brain* [tiab] OR intracrani* [tiab] OR subarachn* [tiab] OR spinal [tiab])) AND

(randomised [tiab] OR randomized [tiab] OR prospective [tiab] OR effectiveness [tiab] OR efficacy [tiab] OR cost-effectiveness [tiab])

AND

(Therapy/Broad[filter] OR "Therapeutics"[Mesh] OR sclerotherap* [tiab] OR surg* [tiab] OR laser* [tiab] OR therapy* [tiab] OR embolisation* [tiab] OR embolization* [tiab] OR injection* [tiab] OR compressi* [tiab] OR endovasc* [tiab])

OR

(questionnaire*[tiab] OR measure*[tiab] OR method*[tiab] OR instrument*[tiab] OR assessment*[tiab] OR tool*[tiab] OR outcome*[tiab] OR index*[tiab] OR indices[tiab] OR profile*[tiab] OR scale*[tiab] OR survey*[tiab] OR patient-reported[tiab] OR physician-reported[tiab])

**EMBASE (14-12-2016)**

(Artery dysplasia OR lymphatic malformation OR lymphangioma OR (vascular malformation* OR vascular anomal* OR arteriovenous malformation* OR vascular malformations OR lymphatic abnormal* OR lymphatic malformation* OR lymphangioma* OR venous malformation* OR venous angioma OR venolymphatic malformation*).ti,ab NOT (cerebel* OR cerebr* OR brain* OR intracrani* OR subarachn* OR spinal)).ti,ab

AND

(randomized OR randomized OR prospective OR effectiveness OR efficacy OR cost-effectiveness).tia,ab

AND

(Exp therapy OR (sclerotherapy* OR surg* OR laser* OR therapy* OR embolization* OR injection* OR compressi* OR endovasc*) OR exp “clinical trial (topic)” OR clinical trial OR randomization OR (clinical and trial) OR random* OR trial OR (questionnaire* or measure* or method* or instrument* or assessment* or tool* or outcome* or index* or indices or profile* or scale* or survey* or patient-reported or physician-reported)).ti,ab

**Search string Search II**

**MEDLINE (20-01-2017)**

("Vascular Malformations"[Mesh] OR vascular malformation* [tiab] OR vascular anomal* [tiab] OR arteriovenous malformation* [tiab] OR AVM[tiab] OR “Lymphatic Abnormalities”[Mesh] OR Lymphatic abnormal*[tiab] OR lymphatic malformation*[tiab] OR “Lymphangioma”[Mesh] OR lymphangioma*[tiab] OR venous malformation*[tiab] OR venous angioma[tiab] OR venolymphatic malformation* [tiab] NOT (cerebel*[tiab] OR cerebr* [tiab] OR brain* [tiab] OR intracrani* [tiab] OR subarachn* [tiab] OR spinal [tiab] OR telangiectasia*[tiab] OR HHT[tiab]))

AND

(instrumentation[sh] OR methods[sh] OR Validation Studies[pt] OR Comparative Study[pt] OR ‘‘psychometrics’’ [MeSH] OR psychometr*[tiab] OR clinimetr*[tw] OR clinometr*[tw] OR ‘‘outcome assessment (health care)’’[MeSH] OR outcome assessment[tiab] OR outcomemeasure*[tw]’’ OR observer variation’’[MeSH] OR observer variation[tiab] OR ‘‘Health Status Indicators’’[Mesh] OR ‘‘reproducibility of results’’[MeSH] OR reproducib*[tiab] OR ‘‘discriminant analysis’’[MeSH] OR reliab*[tiab] OR unreliab*[tiab] OR valid*[tiab] OR coefficient[tiab] OR homogeneity[tiab] OR homogeneous[tiab] OR ‘‘internal consistency’’[tiab] OR (cronbach*[tiab] AND (alpha[tiab] OR alphas[tiab])) OR (item[tiab] AND (correlation*[tiab] OR selection*[tiab] OR reduction*[tiab])) OR agreement[tiab] OR precision[tiab] OR imprecision[tiab] OR ‘‘precise values’’[tiab] OR test-retest[tiab] OR (test[tiab] AND retest[tiab]) OR (reliab* [tiab] AND (test[tiab] OR retest[tiab])) OR stability[tiab] OR interrater[tiab] OR inter-rater[tiab] OR intrarater[tiab] OR intra-rater[tiab] OR intertester[tiab] OR inter-tester[tiab] OR intratester[tiab] OR intra-tester[tiab] OR interobserver[tiab] OR inter-observer[tiab] OR intraobserver[tiab] OR intra-observer[tiab] OR intertechnician[tiab] OR inter-technician[tiab] OR intratechnician[tiab] OR intra-technician[tiab] OR interexaminer[tiab] OR inter-examiner[tiab] OR intraexaminer[tiab] OR intra-examiner[tiab] OR interassay[tiab] OR inter-assay[tiab] OR intraassay[tiab] OR intra-assay[tiab] OR interindividual[tiab] OR inter-individual[tiab] OR intraindividual[tiab] OR intra-individual[tiab] OR interparticipant [tiab] OR inter-participant[tiab] OR intraparticipant[tiab] OR intra-participant[tiab] OR kappa[tiab] OR kappa’s[tiab] OR kappas[tiab] OR repeatab*[tiab] OR ((replicab*[tiab] OR repeated[tiab]) AND (measure[tiab] OR measures[tiab] OR findings[tiab] OR result[tiab] OR results[tiab] OR test[tiab] OR tests[tiab])) OR generaliza*[tiab] OR generalisa*[tiab] OR concordance[tiab] OR (intraclass[tiab] AND correlation*[tiab]) OR discriminative[tiab] OR ‘‘known group’’[tiab] OR factor analysis[tiab] OR factor analyses[tiab] OR dimension*[tiab] OR subscale*[tiab] OR (multitrait[tiab] AND scaling[tiab] AND (analysis[tiab] OR analyses[tiab])) OR item discriminant[tiab] OR interscale correlation*[tiab] OR error[tiab] OR errors[tiab] OR ‘‘individual variability’’[tiab] OR (variability[tiab] AND (analysis[tiab] OR values[tiab])) OR (uncertainty[tiab] AND (measurement[tiab] OR measuring[tiab])) OR ‘‘standard error of measurement’’[tiab] OR sensitiv*[tiab] OR responsive*[tiab] OR ((minimal[tiab] OR minimally[tiab] OR clinical[tiab] OR clinically[tiab]) AND (important[tiab] OR significant[tiab] OR detectable[tiab]) AND (change[tiab] OR difference[tiab])) OR (small*[tiab] AND (real[tiab] OR detectable[tiab]) AND (change[tiab] OR difference[tiab])) OR meaningful change [tiab] OR ‘‘ceiling effect’’[tiab] OR ‘‘floor effect’’[tiab] OR ‘‘Item response model’’[tiab] OR IRT[tiab] OR Rasch[tiab] OR ‘‘Differential item functioning’’[tiab] OR DIF[tiab] OR ‘‘computer adaptive testing’’[tiab] OR ‘‘item bank’’[tiab] OR ‘‘cross-cultural equivalence’’[tiab])

NOT

(“addresses”[Publication Type] OR “biography”[Publication Type] OR “case reports”[Publication Type] OR “comment”[Publication Type] OR “directory”[Publication Type] OR “editorial”[Publication Type] OR “festschrift”[Publication Type] OR “interview”[Publication Type] OR “lectures”[Publication Type] OR “legal cases”[Publication Type] OR “legislation”[Publication Type] OR “letter”[Publication Type] OR “news”[Publication Type] OR “newspaper article”[Publication Type] OR “patient education handout”[Publication Type] OR “popular works”[Publication Type] OR “congresses”[Publication Type] OR “consensus development conference”[Publication Type] OR “consensus development conference, nih”[Publication Type] OR “practice guideline”[Publication Type]) NOT (“animals”[MeSH Terms] NOT “humans”[MeSH Terms])

**EMBASE (20-01-2017)**

(artery dysplasia/ OR lymphatic malformation/ OR lymphangioma/ OR (vascular malformation* or vascular anomal* or arteriovenous malformation* or AVM or Lymphatic abnormal* or lymphatic malformation* or lymphangioma* or venous malformation* or venous angioma or venolymphatic malformation*)).ti,ab

AND

'intermethod comparison'/exp or 'data collection method'/exp or 'validation study'/exp or 'feasibility study'/exp or 'pilot study'/exp or 'psychometry'/exp or 'reproducibility'/exp or reproducib*.ti,ab. or 'audit'.ti,ab. or psychometr*.ti,ab. or clinimetr*.ti,ab. or clinometr*.ti,ab. or 'observer variation'/exp or 'observer variation'.ti,ab. or 'discriminant analysis'/exp or 'validity'/exp or reliab*.ti,ab. or valid*.ti,ab. or 'coefficient'.ti,ab. or 'internal consistency'.ti,ab. or (cronbach* and ('alpha' or 'alphas')).ti,ab. or 'item correlation'.ti,ab. or 'item correlations'.ti,ab. or 'item selection'.ti,ab. or 'item selections'.ti,ab. or 'item reduction'.ti,ab. or 'item reductions'.ti,ab. or 'agreement'.ti,ab. or 'precision'.ti,ab. or 'imprecision'.ti,ab. or 'precise values'.ti,ab. or 'test-retest'.ti,ab. or ('test' and 'retest').ti,ab. or (reliab* and ('test' or 'retest')).ti,ab. or 'stability'.ti,ab. or 'interrater'.ti,ab. or 'inter-rater'.ti,ab. or 'intrarater'.ti,ab. or 'intra-rater'.ti,ab. or 'intertester'.ti,ab. or 'inter-tester'.ti,ab. or 'intratester'.ti,ab. or 'intratester'.ti,ab. or 'interobeserver'.ti,ab. or 'inter-observer'.ti,ab. or 'intraobserver'.ti,ab. or 'intraobserver'.ti,ab. or 'intertechnician'.ti,ab. or 'inter-technician'.ti,ab. or 'intratechnician'.ti,ab. or 'intratechnician'.ti,ab. or 'interexaminer'.ti,ab. or 'inter-examiner'.ti,ab. or 'intraexaminer'.ti,ab. or 'intraexaminer'.ti,ab. or 'interassay'.ti,ab. or 'inter-assay'.ti,ab. or 'intraassay'.ti,ab. or 'intra-assay'.ti,ab. or 'interindividual'.ti,ab. or 'inter-individual'.ti,ab. or 'intraindividual'.ti,ab. or 'intra-individual'.ti,ab. or 'interparticipant'.ti,ab. or 'inter-participant'.ti,ab. or 'intraparticipant'.ti,ab. or 'intraparticipant'.ti,ab. or 'kappa'.ti,ab. or 'kappas'.ti,ab. or 'coefficient of variation'.ti,ab. or repeatab*.ti,ab. or ((replicab* or 'repeated') and ('measure' or 'measures' or 'findings' or 'result' or 'results' or 'test' or 'tests')).ti,ab. or generaliza*.ti,ab. or generalisa*.ti,ab. or 'concordance'.ti,ab. or ('intraclass' and correlation*).ti,ab. or 'discriminative'.ti,ab. or 'known group'.ti,ab. or 'factor analysis'.ti,ab. or 'factor analyses'.ti,ab. or 'factor structure'.ti,ab. or 'factor structures'.ti,ab. or 'dimensionality'.ti,ab. or subscale*.ti,ab. or 'multitrait scaling analysis'.ti,ab. or 'multitrait scaling analyses'.ti,ab. or 'item discriminant'.ti,ab. or 'interscale correlation'.ti,ab. or 'interscale correlations'.ti,ab. or (('error' or 'errors') and (measure* or correlat* or evaluat* or 'accuracy' or 'accurate' or 'precision' or 'mean')).ti,ab. or 'individual variability'.ti,ab. or 'interval variability'.ti,ab. or 'rate variability'.ti,ab. or 'variability analysis'.ti,ab. or ('uncertainty' and ('measurement' or 'measuring')).ti,ab. or 'standard error of measurement'.ti,ab. or sensitiv*.ti,ab. or responsive*.ti,ab. or ('limit' and 'detection').ti,ab. or 'minimal detectable concentration'.ti,ab. or interpretab*.ti,ab. or (small* and ('real' or 'detectable') and ('change' or 'difference')).ti,ab. or 'meaningful change'.ti,ab. or 'minimal important change'.ti,ab. or 'minimal important difference'.ti,ab. or 'minimally important change'.ti,ab. or 'minimally important difference'.ti,ab. or 'minimal detectable change'.ti,ab. or 'minimal detectable difference'.ti,ab. or 'minimally detectable change'.ti,ab. or 'minimally detectable difference'.ti,ab. or 'minimal real change'.ti,ab. or 'minimal real difference'.ti,ab. or 'minimally real change'.ti,ab. or 'minimally real difference'.ti,ab. or 'ceiling effect'.ti,ab. or 'floor effect'.ti,ab. or 'item response model'.ti,ab. or 'irt'.ti,ab. or 'rasch'.ti,ab. or 'differential item functioning'.ti,ab. or 'dif'.ti,ab. or 'computer adaptive testing'.ti,ab. or 'item bank'.ti,ab. or 'cross-cultural equivalence'.ti,ab.

**Search string Search III**

**MEDLINE (22-05-2017)**

(FACT-G*[tiab] OR FACTG*[tiab] OR (Functional assessment*[tiab] AND cancer therap* [tiab] AND general[tiab]) OR PedsQL*[tiab] OR Peds-QL*[tiab] OR “Pediatric quality of life”[tiab] OR "paediatric quality"[tiab] OR “Paediatric QoL”[tiab] OR “Pediatric QoL”[tiab] OR FACIT[tiab] OR “Functional assessment of cancer therapy”[tiab]  OR ("functional assessment"[tiab] AND "chronic illness therapy"[tiab]) OR CIVIQ [tiab] OR CIVIQ2* [tiab] OR “chronic venous insufficiency quality of life”[tiab] OR "chronic venous insufficiency questionnaire"[tiab] OR EQ-5D*[tiab] OR EQ5D*[tiab] OR EuroQol*[tiab] OR Euro-QoL*[tiab] OR “Euro Quality of Life”[tiab] OR SF36*[tiab] OR SF-36*[tiab] OR Short-form-36*[tiab] OR Shortform-36* [tiab] OR shortform36*[tiab] OR SF10*[tiab] OR SF-10*[tiab] OR “Short Form”[tiab] OR shortform [tiab]))

AND

"Lymphatic System"[Mesh:NoExp] OR "Lymphatic Diseases"[Mesh:NoExp] OR "Lymphangitis"[Mesh] OR "Lymphatic Abnormalities"[Mesh:noexp] OR “Lymphangiectasis, Intestinal”[mesh] OR “Lymphedema”[mesh] OR “Lymphocele”[mesh] OR “Mucocutaneous Lymph Node Syndrome”[mesh] OR “Pseudolymphoma”[mesh] OR "Lymphatic Vessel Tumors"[Mesh:NoExp] OR “Lymphangioma”[Mesh] OR lymphatic*[tiab] OR lymphangit*[tiab] OR lymphangiectas*[tiab] OR lymphedem*[tiab] OR lymphoedem*[tiab] OR “Milroy disease”[tiab] OR “Milroy’s disease”[tiab] OR “Nonne Milroy”[tiab] OR Gorham-Stout [tiab] OR lymphocel*[tiab] OR lymphocoel*[tiab] OR mucocutaneous lymph node syndrom*[tiab] OR pseudolymphom*[tiab] OR pseudo-lymphom*[tiab] OR lymphocytom*[tiab] OR lymphoid hyperplas*[tiab] OR lymphangiom*[tiab] OR lymphangioendotheliom*[tiab] OR lymphangio-endotheliom*[tiab] OR "Soft Tissue Neoplasms"[Mesh:NoExp] OR “Vascular Neoplasms”[Mesh] OR "Neoplasms, Connective and Soft Tissue"[Mesh:NoExp] OR "Neoplasms, Adipose Tissue"[Mesh] OR "Lipomatosis"[Mesh] OR "Skin Abnormalities"[Mesh:NoExp] OR “Prolidase Deficiency”[Mesh] OR "Pseudoxanthoma Elasticum"[Mesh] OR "Skin Diseases, Genetic"[Mesh:NoExp] OR "Skin Neoplasms"[Mesh] OR “Facial Neoplasms”[Mesh] OR “Angiolipoma”[Mesh] OR "Neoplastic Syndromes, Hereditary"[Mesh:noexp] OR "Exostoses, Multiple Hereditary"[mesh] OR "Neurofibromatoses"[Mesh] OR soft tissue*[tiab] OR connective tissue*[tiab] OR vascular neoplasm*[tiab] OR vascular tum*[tiab] OR adipose tissue*[tiab] OR angiolipom*[tiab] OR lipom*[tiab] OR fatty tumor*[tiab] OR hibernom*[tiab] OR lipodystrop*[tiab] OR lipo-dystrop*[tiab] OR adiposis[tiab] OR Madelung diseas*[tiab] OR Madelung’s diseas*[tiab] OR Madelung neck[tiab] OR skin abnormalit*[tiab] OR prolidase deficien*[tiab] OR pseudoxanthom*[tiab] OR skin neoplasm*[tiab] OR skin tumor*[tiab] OR skin tumour*[tiab] OR exostos*[tiab] OR neurofibro*[tiab] OR fibroma*[tiab] OR hereditary neoplastic syndrome*[tiab] OR ((localized[tiab] OR localised[tiab] OR skin*[tw] OR cutaneous*[tw] OR dermal[tw] OR dermatol*[tw]) AND scleros*[tiab]) OR "Vascular Diseases"[Mesh:NoExp] OR “Angiomatosis”[Mesh] OR "Telangiectasia, Hereditary Hemorrhagic"[Mesh] OR "Peripheral Vascular Diseases"[Mesh:NoExp] OR "Erythromelalgia"[Mesh] OR "Livedo Reticularis"[Mesh] OR "May-Thurner Syndrome"[Mesh] OR "Phlebitis"[Mesh] OR "Raynaud Disease"[Mesh] OR "Varicose Veins"[Mesh] OR “Venous Insufficiency”[Mesh] OR "Venous Thrombosis"[Mesh:NoExp] OR "Upper Extremity Deep Vein Thrombosis"[Mesh] OR "Venous Thromboembolism"[Mesh] OR “Port-Wine Stain”[Mesh] OR "Neoplasms, Vascular Tissue"[Mesh] OR angioma*[tiab] OR klippel trenaun*[tiab] OR KTW syndrom*[tiab] OR sturge weber[tiab] OR sturge syndrom*[tiab] OR Sturge's Syndrome* [tiab] OR sturge diseas*[tiab] OR peripheral angiopath*[tiab] OR telangiectas*[tiab] OR HHT[tiab] OR Osler diseas* [tiab] OR Osler's Disease[tiab] OR “Osler Weber”[tiab] OR “Weber Osler”[tiab] OR peripheral vascular diseas*[tiab] OR erythromelal*[tiab] OR erythermal*[tiab] OR livedo reticular*[tiab] OR may thurner syndr*[tiab] OR iliocaval compress*[tiab] OR ilio-caval compress*[tiab] OR phlebit*[tiab] OR periphleb*[tiab] OR postphleb*[tiab] OR thrombophlebit*[tiab] OR (inflammat* AND (vein* OR venous[tiab])) OR Raynaud* [tiab] OR varicos*[tiab] OR varix*[tiab] OR varices[tiab] OR vein[tiab] OR venous[tiab] OR venous insufficien*[tiab] OR postthrombotic syndrome*[tiab] OR post-thrombotic syndrome*[tiab] OR venous thromb*[tiab] OR deep vein thromb*[tiab] OR DVT[tiab] OR phlebothrombos*[tiab] OR phlebo-thrombos*[tiab] OR vascular abnormal*[tiab] OR port-wine[tiab] OR portwine[tiab] OR PWS[tiab] OR capillary malformat*[tiab] OR capillary abnormal*[tiab] OR nevus flammeus[tiab] OR naevus flammeus[tiab] OR vascular nev*[tiab] OR vascular neoplasm*[tiab] OR vascular tissue neoplasm*[tiab] OR vessel tumor*[tiab] OR angiofibrom*[tiab] OR hemangiom*[tiab] OR haemangiom*[tiab] OR birthmark*[tiab] OR birth-mark*[tiab] OR (("Lower Extremity"[Mesh:noexp] OR "Leg"[Mesh] OR leg[tiab] OR legs[tiab] OR lower extremit*[tiab] OR lower limb*[tiab]) AND ("Veins"[Mesh:NoExp] OR "Edema"[Mesh:NoExp] OR edem*[tiab] vein*[tiab] OR venous[tiab] )) OR chronic venous diseas*[tiab] OR chronic venous disorder*[tiab] OR (("Veins"[Mesh:NoExp] OR vein*[tiab] OR venous[tiab]) AND ("Ulcer"[Mesh] OR ulcer*[tiab] OR "Stockings, Compression"[Mesh] OR stocking*[tiab]))

AND

(((“reproducibility of results”[MeSH Terms] OR reproducib*[tiab] OR “psychometrics”[MeSH] OR psychometr*[tiab] OR clinimetr*[tiab] OR clinometr*[tiab] OR “observer variation”[MeSH] OR observer variation[tiab] OR “discriminant analysis”[MeSH] OR reliab*[tiab] OR valid*[tiab] OR coefficient[tiab] OR “internal consistency”[tiab] OR (cronbach*[tiab] AND (alpha[tiab] OR alphas[tiab])) OR “item correlation”[tiab] OR “item correlations”[tiab] OR “item selection”[tiab] OR “item selections”[tiab] OR “item reduction”[tiab] OR “item reductions”[tiab] OR agreement[tw] OR precision[tw] OR imprecision[tw] OR “precise values”[tw] OR test–retest[tiab] OR (test[tiab] AND retest[tiab]) OR (reliab*[tiab] AND (test[tiab] OR retest[tiab])) OR stability[tiab] OR interrater[tiab] OR inter-rater[tiab] OR intrarater[tiab] OR intra-rater[tiab] OR intertester[tiab] OR inter-tester[tiab] OR intratester[tiab] OR intra-tester[tiab] OR interobserver[tiab] OR inter-observer[tiab] OR intraobserver[tiab] OR intra-observer[tiab] OR intertechnician[tiab] OR inter-technician[tiab] OR intratechnician[tiab] OR intra-technician[tiab] OR interexaminer[tiab] OR inter-examiner[tiab] OR intraexaminer[tiab] OR intra-examiner[tiab] OR interassay[tiab] OR inter-assay[tiab] OR intraassay[tiab] OR intra-assay[tiab] OR interindividual[tiab] OR inter-individual[tiab] OR intraindividual[tiab] OR intra-individual[tiab] OR interparticipant[tiab] OR inter-participant[tiab] OR intraparticipant[tiab] OR intra-participant[tiab] OR kappa[tiab] OR kappa’s[tiab] OR kappas[tiab] OR “coefficient of variation”[tiab] OR repeatab*[tw] OR ((replicab*[tw] OR repeated[tw]) AND (measure[tw] OR measures[tw] OR findings[tw] OR result[tw] OR results[tw] OR test[tw] OR tests[tw])) OR generaliza*[tiab] OR generalisa*[tiab] OR concordance[tiab] OR (intraclass[tiab] AND correlation*[tiab]) OR discriminative[tiab] OR “known group”[tiab] OR “factor analysis”[tiab] OR “factor analyses”[tiab] OR “factor structure”[tiab] OR “factor structures”[tiab] OR dimensionality[tiab] OR subscale*[tiab] OR “multitrait scaling analysis”[tiab] OR “multitrait scaling analyses”[tiab] OR “item discriminant”[tiab]OR “interscale correlation”[tiab] OR “interscale correlations”[tiab] OR ((error[tiab] OR errors[tiab]) AND (measure*[tiab] OR correlat*[tiab] OR evaluat*[tiab] OR accuracy[tiab] OR accurate[tiab] OR precision[tiab] OR mean[tiab])) OR “individual variability”[tiab] OR “interval variability”[tiab] OR “rate variability”[tiab] OR “variability analysis”[tiab] OR (uncertainty[tiab] AND (measurement[tiab] OR measuring[tiab])) OR “standard error of measurement”[tiab] OR sensitiv*[tiab] OR responsive*[tiab] OR (limit[tiab] AND detection[tiab]) OR “minimal detectable concentration”[tiab] OR interpretab*[tiab] OR (small*[tiab] AND (real[tiab] OR detectable[tiab]) AND (change[tiab] OR difference[tiab])) OR “meaningful change”[tiab] OR “minimal important change”[tiab] OR “minimal important difference”[tiab] OR “minimally important change”[tiab] OR “minimally important difference”[tiab] OR “minimal detectable change”[tiab] OR “minimal detectable difference”[tiab] OR “minimally detectable change”[tiab] OR “minimally detectable difference”[tiab] OR “minimal real difference”[tiab] OR ((real change*[tiab] OR real differenc*[tiab]) AND minimal*[tiab]) OR “ceiling effect”[tiab] OR “floor effect”[tiab] OR “Item response model”[tiab] OR IRT[tiab] OR Rasch[tiab] OR “Differential item functioning”[tiab] OR DIF[tiab] OR “computer adaptive testing”[tiab] OR “item bank”[tiab] OR “cross-cultural equivalence”[tiab]))) NOT (((“addresses”[Publication Type] OR “biography”[Publication Type] OR “case reports”[Publication Type] OR “comment”[Publication Type] OR “directory”[Publication Type] OR “editorial”[Publication Type] OR “festschrift”[Publication Type] OR “interview”[Publication Type] OR “lectures”[Publication Type] OR “legal cases”[Publication Type] OR “legislation”[Publication Type] OR “letter”[Publication Type] OR “news”[Publication Type] OR “newspaper article”[Publication Type] OR “patient education handout”[Publication Type] OR “popular works”[Publication Type] OR “congresses”[Publication Type] OR “consensus development conference”[Publication Type] OR “consensus development conference, nih”[Publication Type] OR “practice guideline”[Publication Type]) NOT (“animals”[MeSH Terms] NOT “humans”[MeSH Terms])))

**EMBASE (22-05-2017)**

(FACT-G or FACTG or FACT-G7 or FACTG7 or FACT-general or FACTgeneral or FACTgeneric or FACT-generic or FACT-GP or FACIT or (functional assessment adj3 (chronic illness or cancer therap*)) or PedsQL* or ((Peds or p?ediatric) adj (Qol or QL* or quality of life)) or CIVIQ or CIVIQ2* or CIVI-Q or CIVI-Q2* or ((chronic venous insufficien* or CIV or CIVI) adj (Qol or QL or quality of life or questionnaire*)) or EQ-5D* or EQ5D* or EuroQol* or Euro-QoL* or "Euro Quality of Life" or SF36* or ((SF or short-form or shortform) adj2 36*) or shortform36* or SF10* or SF-10*).tw,kw.

AND

(lymphatic system/ or lymph vessel/ or lymph vessel endothelium/ or lymphatic system disease/ or intestine lymphangiectasia/ or lymphangiectasis/ or lymphangitis/ or exp lymphedema/ or lymphocele/ or lymphoid hyperplasia/ or mucocutaneous lymph node syndrome/ or pseudolymphoma/ or lymphangioma/ or (lymphatic* or lymphangit* or lymphangiectas* or lymph?edem* or Milroy* disease or Nonne-Milroy or Gorham-Stout or lymphoc?el* or mucocutaneous lymph node syndrom* or Kawasaki or pseudolymphom* or pseudo-lymphom* or lymphocytom* or lymphoid hyperplas* or lymphangiom* or lymphangioendotheliom* or lymphangio-endotheliom*).tw,kw.

OR

Soft tissue tumor/ or angiolipoma/ or connective tissue tumor/ or solitary fibrous tumor/ or subcutaneous tissue tumor/ or vascular tumor/ or angioma/ or hemangioendothelioma/ or hemangiofibroma/ or capillary hemangioma/ or hemangiomatosis/ or exp skin hemangioma/ or nevus flammeus/ or connective tissue disease/ or lipedema/ or lipomatosis/ or congenital skin disease/ or cutis marmorata telangiectatica congenita/ or prolidase deficiency/ or connective tissue diseases affecting the skin/ or hereditary connective tissue disease/ or skin disease/ or skin blood vessel disorder/ or exp skin hemangioma/ or skin tumor/ or benign skin tumor/ or fibroadenoma/ or exp fibroma/ or exp adenofibroma/ or cystadenofibroma/ or subcutaneous nodule/ or face tumor/ or hereditary multiple exostosis/ or exp neurofibromatosis/ or neurofibromatosis type 1/ or neurofibromatosis type 2/ or (soft tissue* or connective tissue* or vascular neoplasm* or vascular tum* or adipose tissue* or angiolipom* or lipom* or fatty tumor* or hibernom* or lipodystrop* or lipo-dystrop* or adiposis or Madelung diseas* or Madelung* diseas* or Madelung neck or skin abnormalit* or prolidase deficien* or pseudoxanthom* or skin neoplasm* or skin tumor* or skin tumour* or exostos* or neurofibro* or fibroma* or hereditary neoplastic syndrome*).tw,kw

OR

vascular disease/ or endothelial dysfunction/ or endothelium lesion/ or peripheral vascular disease/ or angiolipoma/ or artery disease/ or artery lesion/ or peripheral blood vessel malformation/ or arteriovenous malformations/ or artery malformation/ or vein malformation/ or Raynaud phenomenon/ or telangiectasia/ or cutis marmorata telangiectatica congenital/ or Rendu Osler Weber disease/ or vasculitis/ or exp phlebitis/ or small vessel vasculitis/ or systemic vasculitis/ or vein disease/ or varicosis/ or leg varicosis/ or vein insufficiency/ or chronic vein insufficiency/ or vein thrombosis/ or deep vein thrombosis/ or venous thromboembolism/ or leg thrombophlebitis/ or lower extremity deep vein thrombosis/ or superficial thrombophlebitis or thrombophlebitis/ or upper extremity deep vein thrombosis/ or thromboembolism/ or thrombosis/ or leg thrombosis/ or postoperative thrombosis/ or thrombus/ or vascular fragility/ or vascular lesion/ or vascular tumor/ or angioma/ or hemangioma/ or capillary hemangioma/ or hemangiomatosis/ or exp skin hemangioma/ or exp lymphangioma/ or hemangioendothelioma/ or hemangiofibroma/ or exp hemangiomatosis/ or erythromelalgia/ or livedo reticularis/ or exp nevus flammeus/ or (klippel trenaun* or KTW syndrom* or sturge weber or sturge syndrom* or Sturge* Syndrome* or sturge diseas* or peripheral angiopath* or telangiectas* or HHT or Osler diseas* or Osler* Disease or Osler Weber or Weber Osler or peripheral vascular diseas* or erythromelal* or erythermal* or livedo reticular* or may thurner syndr* or iliocaval compress* or ilio-caval compress* or cockett or phlebit* or periphleb* or postphleb* or thrombophlebit* or (inflammat* and (vein* or venous)) or Raynaud* or varicos* or varix* or varices or venous insufficien* or postthrombotic syndrome* or post-thrombotic syndrome* or venous thromb* or deep vein thromb* or DVT or phlebothrombos* or phlebo-thrombos* or vascular abnormal* or port-wine or capillary malformat* or capillary abnormal* or nevus flammeus or naevus flammeus or vascular nev* or vascular neoplasm* or vascular tissue neoplasm* or vessel tumor* or angiofibrom* or hemangiom* or haemangiom* or birthmark* or birth-mark* or ((leg OR legs or lower extermit* or lower limb*) and (edem* or vein* or venous)) OR chronic venous diseas* or chronic venous disorder* or ((vein* or venous) and (ulcer* or stocking*))).tw,kw.)

AND

exp intermethod comparison/ or exp data collection method/ or exp validation study/ or exp feasibility study/ or exp pilot study/ or exp psychometry/ or exp reproducibility/ or (reproducib* or audit or psychometr* or clinimetr* or clinometr*).tw,kw or exp observer variation/ or observer variation.tw,kw or exp discriminant analysis/ or exp validity/ or (reliab* or valid* or coefficient or internal consistency or (cronbach* and (alpha or alphas)) or item correlation or item correlations or item selection or item selections or item reduction or item reductions or agreement or precision or imprecision or precise values or test-retest or (test and retest) or (reliab* and (test or retest)) or stability or interrater or inter-rater or intrarater or intra-rater or intertester or inter-tester or intratester or intratester or interobeserver or inter-observer or intraobserver or intraobserver or intertechnician or inter-technician or intratechnician or intratechnician or interexaminer or inter-examiner or intraexaminer or intraexaminer or interassay or inter-assay or intraassay or intra-assay or interindividual or inter-individual or intraindividual or intra-individual or interparticipant or inter-participant or intraparticipant or intraparticipant or kappa or kappas or coefficient of variation or repeatab* or (replicab* or repeated and (measure or measures or findings or result or results or test or tests)) or generaliza* or generalisa* or concordance or (intraclass and correlation*) or discriminative or known group or factor analysis or factor analyses or factor structure or factor structures or dimensionality or subscale* or multitrait scaling analysis or multitrait scaling analyses or item discriminant or interscale correlation or interscale correlations or (error or errors and (measure* or correlat* or evaluat* or accuracy or accurate or precision or mean)) or individual variability or interval variability or rate variability or variability analysis or (uncertainty and (measurement or measuring)) or standard error of measurement or sensitiv* or responsive* or (limit and detection) or minimal detectable concentration or interpretab* or (small* and (real or detectable) and (change or difference)) or meaningful change or minimal important change or minimal important difference or minimally important change or minimally important difference or minimal detectable change or minimal detectable difference or minimally detectable change or minimally detectable difference or minimal real change or minimal real difference or minimally real change or minimally real difference or ceiling effect or floor effect or item response model or irt or rasch or differential item functioning or dif or computer adaptive testing or item bank or cross-cultural equivalence).tw,kw.
