# Supplementary material for: Outcome measurement instruments for peripheral vascular malformations and an assessment of the measurement properties: a systematic review
Source: Qual Life Res. 2019 Sep 23;29(1):1–17. doi: 10.1007/s11136-019-02301-x (PMC6962285; doi:10.1007/s11136-019-02301-x)
Supplement: Supplementary file 5 — Supplementary material 5 (DOCX 20 kb) [file 11136_2019_2301_MOESM5_ESM.docx]

| ***Online Resource 5.*** COSMIN criteria for good measurement properties, as defined by Prinsen et al, 2016^1^, used with permission of the author. | | |
| --- | --- | --- |
| **Measurement property** | **Rating*** | **Criteria** |
| Content validity (including face validity) | + | All items refer to relevant aspects of the construct to be measured AND are relevant for the target population AND are relevant for the context of use AND together comprehensively reflect the construct to be measured |
|  | ? | Not all information for ‘+’ reported |
|  | - | Criteria for ‘+’ not met |
| Structural validity | + | **CTT:**  Unidimensionality: EFA: First factor accounts for at least 20% of the variability AND ratio of the variance explained by the first to the second factor greater than 4 OR Bi-factor model: Standardized loadings on a common factor >0.30 AND correlation between individual scores under a bi-factor and unidimensional model >0.90  Structural validity: CFI or TLI or comparable measure >0.95 AND RMSEA <0.06 OR SRMR <0.08 |
|  |  | **Rasch/IRT:** At least limited evidence for unidimensionality or positive structural validity AND no evidence for violation of local independence: Rasch: standardized item-person fit residuals between -2.5 and 2.5; OR IRT: residual correlations among the items after controlling for the dominant factor < 0.20 OR Q3's < 0.37 AND no evidence for violation of monotonicity: adequate looking graphs OR item scalability >0.30 AND adequate model fit: Rasch: infit and outfit mean squares ≥ 0.5 and ≤ 1.5 OR Z-standardized values > -2 and <2; OR IRT: G2 >0.01; Optional additional evidence: Adequate targeting; Rasch: adequate person-item threshold distribution; IRT: adequate threshold range  No important DIF for relevant subject characteristics (such as age, gender, education), McFadden's R2 < 0.02 |
|  | ? | CTT: Not all information for '+' reported IRT: Model fit not reported |
|  | - | Criteria for ‘+’ not met |
| Internal consistency | + | At least limited evidence for unidimensionality or positive structural validity AND Cronbach's alpha(s) ≥ 0.70 and ≤ 0.95 |
|  | ? | Not all information for ‘+’ reported OR conflicting evidence for unidimensionality or structural validity OR evidence for lack of unidimensionality or negative structural validity |
|  | - | Criteria for ‘+’ not met |
| Reliability | + | ICC or weighted Kappa ≥ 0.70 |
|  | ? | ICC or weighted Kappa not reported |
|  | - | Criteria for ‘+’ not met |
| Measurement error | + | SDC or LoA < MIC |
|  | ? | MIC not defined |
|  | - | Criteria for ‘+’ not met |
| Hypotheses testing | + | At least 75% of the results are in accordance with the hypotheses |
|  | ? | No correlations with instrument(s) measuring related construct(s) AND no differences between relevant groups reported |
|  | - | Criteria for ‘+’ not met |
| Cross-cultural validity | + | No important differences found between language versions in multiple group factor analysis or DIF analysis |
|  | ? | Multiple group factor analysis AND DIF analysis not performed |
|  | - | One or more criteria for ‘+’ not met |
| Criterion validity | + | Convincing arguments that gold standard is “gold” AND correlation with gold standard ≥ 0.70 |
|  | ? | Not all information for ‘+’ reported |
|  | - | Criteria for ‘+’ not met |
| Responsiveness | + | At least 75% of the results are in accordance with the hypotheses |
|  | ? | No correlations with changes in instrument(s) measuring related construct(s) AND no differences between changes in relevant groups reported |
|  | - | Criteria for ‘+’ not met |
| CTT: classical test theory; EFA: exploratory factor analysis; CFI: comparative fit index; TLI: Tucker-Lewis index; RMSE: root mean square error of approximation; SRMR: standardized root mean residuals; IRT: item response theory; DIF: differential item functioning; ICC: intraclass correlation coefficient; SDC: smallest detectable change, LoA: limits of agreement; MIC: minimal important change; *: “+”= positive rating, “?”= indeterminate rating, “-“= negative rating | | |
|  |  |  |
|  |  |  |

1 Prinsen CA, Vohra S, Rose MR *et al.* How to select outcome measurement instruments for outcomes included in a "Core Outcome Set" - a practical guideline. *Trials* 2016; **17**: 449.
