# Supplementary material for: Outcome measurement instruments for peripheral vascular malformations and an assessment of the measurement properties: a systematic review
Source: Qual Life Res. 2019 Sep 23;29(1):1–17. doi: 10.1007/s11136-019-02301-x (PMC6962285; doi:10.1007/s11136-019-02301-x)
Supplement: Supplementary file 6 — Supplementary material 6 (DOCX 109 kb) [file 11136_2019_2301_MOESM6_ESM.docx]

| ***Online Resource 6, Part A.*** Methodological quality of the included studies according to the COSMIN checklist, and overall results of the measurement properties analyses: reliability, responsiveness and interpretability. | | | | | | | | | | | | | |
| --- | --- | --- | --- | --- | --- | --- | --- | --- | --- | --- | --- | --- | --- |
| **Author, year** | **Reliability** | | | | | | **Responsiveness** | | **Interpretability** | | |  | |
|  |  |  |  |  |  |  |  |  |  | | |  | |
|  | **Internal consistency** | | **Reliability** | | **Measurement error** | | **Responsiveness** | | **Floor and ceiling effects** | | | **Feasibility** | |
|  | Result Cronbach's alpha | COSMIN rating | Result | COSMIN score | Result | COSMIN rating | Result | COSMIN rating |  | | |  | |
| ***The Lymphatic Malformation Function Instrument (LMF)*** | | | | | | | | | | | | | |
| Balakrishnan K, 2012^1^ | - | - | - | - | - | - | - | - | - | | | - | |
| Kirkham EM, 2015^2^ | Signs α = 0,86 Impacts α = 0,84 | Fair | **Test-retest**  ICC coefficients: Signs 0,88 (95% CI, 0,70-0,95) Impacts 0,58 (95% CI, 0,16-0,82) Total 0,82 (95% CI, 0,58-0,93) | Poor | - | - | - | - | Floor effect in 2 items (85% and 83%) of which 1st removed and 2nd retained because of clinical importance. Remaining items also skewed toward floor, therefore reduction of 5-point into 3-point scale. | | | - | |
| ***The 20-Item ChronIc Venous Insufficiency Quality of Life Questionnaire (CIVIQ-20)*** | | | | | | | | | | | | | |
| Launois R, 1996 (1)^3^ | Physical α = 0,830 Psychological impairment α = 0,90 Social impairment α = 0,673 Pain α = 0,832 | Fair | **Pearson's correlation coefficient for item scores on day 0 and 15**  Severity items: 0,8183 - 0,9633 Physical 0,9774 Psychological 0,9630 Social 0,9447 Pain 0,9493 Overall quality of life score 0,9803 Importance items: 0,8504 - 0,9632 Physical 0,9308 Psychological 0,9550 Social 0,8529 Pain 0,9352 Overall quality of life score 0,9512 | Fair | - | - | - | - | - | | | - | |
| Launois R, 1996 (2)^3^ | Physical α = 0,711 Psychological impairment α = 0,853 Social impairment α = 0,654 Pain α = 0,779 | Fair | - | - | - | - | **Pearson's correlation coefficients between QoL change scores and functional symptoms between day 0 and 60:**  Physical ranging 0,2210 - 0,3948, P<0,001 Psychological ranging 0,2489 - 0,3624, P<0,001 Social ranging 0,1986 - 0,3109, P<0,001 Pain ranging 0,3115 - 0,5643, P<0,001 QoL score ranging 0,3165 - 0, 4876, P<0,001 | Poor | - | | | - | |
| Jantet G, 2000^4^ | All α's > 0,70 | Fair | **Test-retest:**  ICCs > 0.80 in all dimensions and global index | Fair | - | - | Parallel evolution of change in clinical criteria and QoL data. | Fair | - | | | - | |
| Jantet G, 2002^5^ | All α's > 0.70 | Poor | **Test-retest:**  All ICCs > 0.80 | Fair | - | - | Changes in CIVIQ-20 score highly significant (P=0,0001) for improved patients, irrespective of the various clinical criteria defined for assessing patients as improved. All effect size ratios < 0.4. | Fair | - | | | - | |
| Lozano FS, 2002^6^ | All α's > 0.70 | Fair | **Test-retest:**  ICC coefficients ranging from 0.87 - 0.92 | Fair | - | - | Paired t-tests highly significant in all domains (p<0.001) in changes from baseline to day 180. **Correlations between QoL change scores and clinical scores:** Psychological ranging 0.17-0.33 Pain ranging 0.17-0.43 Physical ranging 0.10-0.23 Social ranging 0.10-0.28 QoL change scores not correlated with changes in CEAP. | Poor | - | | | - | |
| Erevnidou K, 2004^7^ | Physical α = 0.83 Psychological impairment α = 0.77 Social impairment α = 0.83 Pain α = 0.82 Total α = 0.79 | Fair | **Test-retest:**  Pearson's correlation coefficient 0.98 (0.845 - 0.97) | Fair | - | - | - | - | - | | | - | |
| Launois R, 2010^8^ | Physical α = 0.86 Psychological impairment α = 0.89 Social impairment α = 0.76 Pain α = 0.83 Total α = 0.94 | Good | **Test-retest:** ICC coefficients Stable symptoms ranging 0,9385 - 0,9441 Stable CEAP 0,9319 All parameters together stable 0,9561 | Good | - | - | In clinically improved patients, a parallel increase of CIVIQ-20 scores was found over time after treatment. Mean change score between day 0 and 180; ES: Swelling 21,1; 1,26 Heaviness 20,1; 1,24 Cramps 21,1; 1,29 Pain 29,2; 1,73 | Fair | - | | | - | |
| Ozdemir OC, 2016^9^ | α = 0,93 | Poor | **Test-retest:**  ICC type 2:1 = 0,80 | Fair | SEM = 2,63 MDC = 5,79 | Fair | - | - | - | | | - | |
| Biemans AAM, 2011^10^ | CIVIQ-1 α = 0,94 | Poor | **Test-retest CIVIQ-1-2**  Spearman's ρ = 0,86 **Median global score**  CIVIQ-1: 17,50 (IQR 8,13 - 33,75) CIVIQ-2: 17,50 (IQR 7,50 - 33,75) | Good | - | - | Median CIVIQ score significantly decreased from 18,75 (IQR 11,25 - 33,75) to 12,50 (IQR 5,00 - 22,50) 4 weeks after therapy. Mean global score decreased from 23,68 (SD 16,04) to 15,66 (SD 13,20). **Patients who had EVLA plus phlebectomy vs. EVLA only** Median CIVIQ score significantly decreased from 20,63 (IQR 8,1 - 34,06) to 11,25 (IQR 1,25 -15,93) vs 25,00 (IQR 16,25 - 36,25) to 18,75 (IQR 12,50 - 28,75). **Patients treated bilaterally vs. unilaterally**  Median CIVIQ score decrease from 16,25 (IQR 8,75 - 36,25) to 10,00 (IQR 5 - 22,50) vs. 23,75 (IQR 12,50 - 33,75) to 15,00 (IQR 6,25 - 28,75). | Fair | Ceiling effect in 3 items with >70% of respondents indicating lowest score. No floor effects. | | | 6,2% of patients did not respond to ≥ 3 items. None of the items were considered suboptimal, missing responses 0 - 9,4%. 2 items were missing 8,8 and 9,4% of participants. | |
| ***The Short Form 36 (SF-36) Health Survey*** | | | | | | | | | | | | | |
| Garratt AM, 1993^11^ | PF α = 0,92 PA α = 0,89 BP α = 0,86 GH α = 0,83 V α = 0,86 SF α = 0,80 EWB α = 0,86 MH α = 0,86 | Fair | - | - | - | - | - | - | - | | | - | |
| Garratt AM, 1994^12^ | - | - | - | - | - | - | 1. Relation between change scores in all dimensions of SF-36 and responses to transition question highly significant for 4 patient groups after 1 year. 2. Significant improvements after 1 year in all SF-36 domain scores except for SF for patients with varicose veins compared to general population, corrected for age, sex and socioeconomic status. | Fair | - | | | - | |
| Ruta DA, 1994^13^ | PF α = 0, 92 PA α = 0,89 BP α = 0,86 GH α = 0,83 V α = 0,86 SF α = 0,80 EWB α = 0,86 MH α = 0,86 | Poor | **Test-retest:** Pearson's correlation coefficients: PF α = 0, 93 PA α = 0,76 BP α = 0,82 GH α = 0,88 V α = 0,84 SF α = 0,80 EWB α = 0,66 MH α = 0,81 | Fair | - | - | - | - | - | | | - | |
| Garratt AM, 1996^14^ | - | - | **Test-retest:** ICC coefficients: PF 0,94 PA 0,75 BP 0,84 GH 0,86 V 0,74 SF 0,79 EWB 0,59 MH 0,77 | Fair | - | - | Significant correlation between mean change scores SF-36 dimensions after 1 year and reported transition (much better, better, the same, worse, much worse), except for domain GH. Significantly greater level of improvement in patients admitted to hospital compared to patients not admitted to hospital. | Fair | - |  |  | - |  |
| Walters SJ, 1999^15^ | SF α < 0,7 All other domains α > 0,7 | Poor | - | - | - | - | **1.** **Leg ulcer status at 3-months follow-up** No significant evidence of a differential health change between healed and not healed ulcer groups. **2. Perceived health change at 3-months follow-up** One-way ANOVA: negative or same scores on transition question significantly associated with negative changes in all dimension scores. Positive scores on transition question significantly associated with positive changes in dimension scores in BP, V, EWB and MH, and with negative changes in GHP and SF. **3.** **12-month follow-up** Two-sample t-test: Initial ulcer healed and stayed healed significantly associated with positive changes in pain and MH and negative changes in PA. Ulcer not healed significantly associated with negative changes in PA, BP and MH. | Good | Floor effect in PA and EWB with >20% of respondents indicating lowest score. Ceiling effects in PA, EWB and SF. |  |  | - |  |
| Jull A, 2010 (2)^16^ | - | - | - | - | - | - | **1. SRMs from baseline to 12 weeks** Ulcer healed: domains ranging from -0,07 to 0,47. Ulcer unhealed: domains ranging from -0,03 to 0,24. Both suggesting modest responsiveness to change. **2. Ability to distinguish between healed and unhealed participants at 12 weeks** **using unpaired t tests** Significantly positive mean difference on domains PA, BP and MH, indicating greater increase in perceived health in healed ulcers compared with unhealed ulcers. | Fair | - | | | - | |
| Franks PJ, 2003^17^ | PF α = 0,94 PA α = 0,95 BP α = 0,86 GH α = 0,82 V α = 0,70 SF α = 0,76 EWB α = 0,93 MH α = 0,76 | Poor | - | - | - | - | **1. Mean difference between baseline and after 12 weeks of treatment; paired t-test:**  PF: 2,0, NS PA: -1,5, NS BP: 2,1, NS GH: -0,9, NS V: 0,0, NS SF: -1,7, NS EWB: 1,4, NS MH: -1,6, NS **2. SRM between patients with open vs. closed ulcerations following 12 weeks of treatment:** PF: 0,02 vs. 0,09 PA: 0,27 vs. 0,16 BP: 0,60 vs. -0,09 GH: -0,16 vs. <0,01 V: 0,29 vs. -0,12 SF: 0,07 vs. 0,09 EWB: 0,30 vs. -0,06 MH: 0,29 vs. -0,26 | Fair | Ceiling- and floor effect per domain (%): PF: 2 ; 25 PA: 35 ; 50 BP: 18 ; 5 GH: 3 ; 2 V: 2 ; 3 SF: 22 ; 9 EWB: 51 ; 36 MH: 5 ; 0 | | | - | |
| Franks PJ, 2006 (1)^18^ | PF α = 0,94 PA α = 0,94 BP α = 0,88 GH α = 0,80 V α = 0,83 SF α = 0,85 EWB α = 0,95 MH α = 0,81 | Poor | - | - | - | - | **SRM (95% CI) after 24 weeks of treatment** PF 4,3 (1,0 - 7,6), NS PA 22,9 (12,6 - 33,3) BP 11,4 (6,2 - 16,5) GH -2,4 (-5,7 - 0,8), NS V 0,5 (-3,1 - 4,2), NS SF 8,5 (1,6 - 15,3) EWB 14,4 (5,2 - 23,7) MH 4,6 (1,5 - 7,7) **ANOVA after 24 weeks of treatment** Significantly greater improvements for patients in physical functioning and general health with improved health. | Fair | - | | | - | |
| Qiang cao, 2013^19^ | PF α = 0,812 PA α = 0,881 BP α = 0,901 GH α = 0,820 V α = 0,9000 SF α = 0,839 EWB α = 0,899 MH α = 0,905 Total score α = 0,911 | Excellent | **Test-retest: ICC coefficients**  PF 0,803 PA 0,701 BP 0,711 GH 0,792 V 0,749 SF 0,699 EWB 0,781 MH 0,823 | Good | - | - | **Improvement in mean difference between baseline and 1 month post-treatment dimension scores using paired t test** PF: 40, P<0,01 PA: 24, P<0,01 BP: 28, P<0,01 GH: 15, P<0,01 V: 14, P<0,01 SF: 25, P<0,01 EWB: 48, P<0,01 MH: 14, P<0,01 | Poor | - | | | - | |
| ***The Euro Quality of Life - 5 Domain (EQ-5D)*** | | | | | | | | | | | | | |
| Iglesias CP, 2005^20^ | - | - | - | - | - | - | **ES and SRM from baseline to 3 months** Ulcer healed: 0,5 ; 0,4 Ulcer unhealed: 0,1 ; 0,1 | Fair | - | | | - | |
| Jull A, 2010 (1)^16^ | - | - | - | - | - | - | **1. SRMs from baseline to 12 weeks** Ulcer healed: domains ranging from 0,13 to 0,31. Ulcer unhealed: domains ranging from 0,10 to 0,16. Both suggesting modest responsiveness to change. **2. Ability to distinguish between healed and unhealed participants at 12 weeks** **using unpaired t tests** Insignificant, small positive mean difference, indicating greater increase in perceived health in healed ulcers compared with unhealed ulcers. | Fair | - | | | - | |
| Franks PJ, 2006 (2)^18^ | - | - | - | - | - | - | **SRM (95% CI) after 24 weeks of treatment**: 0,05 (-0,004 - 0,09) **ANOVA after 24 weeks of treatment** EQ-5D improved with better perceived health, no statistical significance (p=0,10) | Fair | No floor and ceiling effects. | | | - | |
| ***Pediatric Quality of Life Inventory Neurofibromatosis Type 1 (PedsQL NF1, adults)*** | | | | | | | | | | | | | |
| Nutakki K, 2013 (1)^21^ | Pilot testing α = 0,82 | Poor | - | - | - | - | - | - | - | | | 4,8% missing responses for all subscales. The Sexual Functioning subscale highest number of missing responses (30,8%) and was not included in any statistical analyses. | |
| Nutakki K, 2013 (2)^21^ | Physical functioning α = 0,93 Emotional functioning α = 0,92 Social functioning α = 0,73 Cognitive functioning α = 0,94 Communication α = 0,84 Worry α = 0,83 Perceived physical appearance α = 0,90 Pain and hurt α = 0,93 Paresthesia’s α = 0,87 Skin irritation α = 0,83 Sensation α = 0,72 Movement and balance α = 0,91 Daily activities α = 0,96 Fatigue α = 0,90 Treatment anxiety α = 0,87 Total score α = 0,97 | Excellent | - | - | - | - | - | - | - | | | - | |
| ***Pediatric Quality of Life Inventory Neurofibromatosis Type 1 (PedsQL NF1, children, adolescents and young adults)*** | | | | | | | | | | | | | |
| Nutakki K, 2017^22^ | - | - | - | - | - | - | - | - | - | | | - | |
| ICC: intraclass correlation; QoL: quality of life; CEAP: comprehensive classification system for chronic venous disorders; ES: effect size; SEM: standard error of the measurement; MDC: minimum detectable change; IQR: interquartile range; PF: physical functioning; PA: physical aspects; BP: bodily pain; GH: general health; V: vitality; SF: social functioning; EWB: emotional well-being; MH: mental health; NS: not significant; ANOVA: analysis of variance; SRM: standardized response mean; - : not in described in article. | | | | | | | | | | | | | |

| ***Online Resource 6, Part B.*** Methodological quality of the included studies according to the COSMIN checklist, and overall results of the measurement properties analyses: validity. | | | | | | | | | | |
| --- | --- | --- | --- | --- | --- | --- | --- | --- | --- | --- |
| **Author, year** | **Validity** | | | | | | | | | |
|  |  |  | **Construct validity** | | | | | | | |
|  | **Content validity** | | **Structural validity** | | **Hypotheses-testing validity** | | | | **Cross-cultural validity** | |
|  | Result | COSMIN rating | Result | COSMIN rating | Hypotheses | Result | COSMIN rating | | Result | COSMIN rating |
| ***The Lymphatic Malformation Function Instrument (LMF)*** | | | | | | | | | | |
| Balakrishnan K, 2012^1^ | Semi-structured interview questions based on clinical and research experience. Responses reviewed and coded for function- or symptom-related domains, coding reconciled between reviewers. Interviewees (patients and parents) rated impact on each domain using 5-point Likert scales. | Excellent | - | - | - | - | - | | - | - |
| Kirkham EM, 2015^2^ | - | - | Two items combined into 1 item due to high interitem correlation (r=0,68). Five items removed due to high interitem correlations (r>0,7) Exploratory PCA with PAF, promax and varimax rotation 2-factor model Eigenvalues 4,96; 1.63 Explained cumulative variance 63%, 84%, 95% Factor 1 (Signs) 7 items, loadings 0,51 - 0,86 Factor 2 (Impacts) 5 items, loadings 0,61 - 0,82 2 items removed due to not loading onto at least 1 factor. | Fair | **Expected correlations:**  Significant positive correlation between instrument scores (total and domains) and stage. Correlation between instrument scores (total and domains) and age or sex. | Spearman's correlation coefficients: Stage: Total ρ = 0,61, P<0,1 Signs ρ = 0,73, P<0,001 Impacts ρ = 0,30, P<0,04 Age: Total and domains P > 0,30 Sex: Total and domains P > 0,10 Source of recruitment: Total and domains P > 0,05 | Fair | | - | - |
| ***The 20-Item ChronIc Venous Insufficiency Quality of Life Questionnaire (CIVIQ-20)*** | | | | | | | | | | |
| Launois R, 1996 (1)^3^ | Process of item selection based on input from specialists, published data and direct definitions of complaints obtained from patients. Use of factorial analysis to identify and exclude redundant items. 99% response rate. 75,6% answered all questions. Non-response rate for each item 1 - 3,9%. | Good | Exploratory PCA with PAF and varimax rotation 4-factor model Eigenvalues 8,30; 1,08; 0,86; 0,69 Proportions of variance explained: 41,50%, 5,40%, 4,30%, 3,50% | Fair | **Expected correlations:** Positive correlation between clinical severity scores and QoL score. | Pearson's correlation coefficients between CIVIQ and clinical scores: **Functional score:**  Physical 0,4571 Psychological 0,4632 Social dimension: 0,3164 Pain dimension: 0,6044 QoL score 0,5243 **Objective score:** Physical 0,4423 Psychological 0,3788 Social dimension: 0,3321 Pain dimension: 0,4005 QoL score 0,4499 | Poor | | - | - |
| Launois R, 1996 (2)^3^ | - | - | Exploratory PCA with PAF and varimax rotation 4-factor model Eigenvalues 6,59; 1,25; 0,81; 0,69 Proportions of variance explained: 33,0%, 6,30%, 4,10%, 3,50% | Fair | - | - | - | | - | - |
| Jantet G, 2000^4^ | - | - | **Exploratory PAF on 20 items:**  4-factor model **MAP on 20 items:**  All item-scale correlations > 0,40. Scaling success good for psychological and pain dimension and acceptable for physical dimension. | Fair | **Expected correlations:** 1. Positive item-scale correlations. 2. Positive correlations between clinical criteria and QoL scores at baseline. | **Correlations between clinical criteria and QoL scores at baseline:** Excellent and highly significant (p<0,0001) | Poor | | All items translated into Czech, Slovak, Hungarian, Polish, Russian and Spanish | Poor |
| Jantet G, 2002^5^ | - | - | All item-scale correlations > 0.4 | Poor | No hypotheses and expected correlations not stated | Significantly different CIVIQ score in all domains when stratified according to CEAP classification for severity of illness (p=0.0001). Significantly different CIVIQ score between group with and without venous reflux (p=0.0001) | Fair | | - | - |
| Lozano FS, 2002^6^ | - | - | - | - | - | - | - | | All items translated into Spanish language. | Poor |
| Erevnidou K, 2004^7^ | - | - | Exploratory PCA with varimax rotation 4-factor model with eigenvalues > 1.00 Proportions of variance explained: 27%, 21%, 12%, 7% | Poor | No hypotheses and expected correlations not stated | Pearson's correlation coefficients between CIVIQ and SF-36: Physical dimension: -0.82 Psychological dimension: -0.48 Social dimension: 0.54 Pain dimension: 0.77 | Fair | | All items translated into Greek language using multiple forward and single backward translations. | Poor |
| Launois R, 2010^8^ | - | - | Exploratory PAF with promax rotation 4-factor model Proportion of variance explained: 57% Czech Republic and Poland: factor 1 (physical), 2 (psychological), 3 (pain), 4 (uninterpretable) Spain: factors uninterpretable | Good | **Expected correlations:** Negatively correlated in stable patients with known differences in symptoms scores and CEAP classes at baseline. | Spearman's correlation coefficients between CIVIQ-20 scores and stable patients with known differences in symptom scores and CEAP classes: Swelling: -0.38561 Heaviness: -0.41706 Cramps: -0.33244 CEAP: -0.22032 | Good | | - | - |
| Ozdemir OC, 2016^9^ | - | - | Exploratory FA with MLE and varimax rotation 3-factor model identified by scree plot. Total variance explained: 56,32% | Fair | **Expected correlations:**  Positive correlation between CIVIQ-20 and VEINES-QoL and NHP. | Pearson's correlation coefficients between CIVIQ-20 and VEINES-QoL and NHP: VEINES-QoL1: r=-0,574; P<0,00 VEINES-QoL2: r=-0,592; P=0,00 NHP1 r=0,770; P<0,00 NHP2: r=0,7000; P<0 | Fair | | All items translated into Turkish language using multiple forward and backward translations. | Fair |
| Biemans AAM, 2011^10^ | - | - | Exploratory PAF with promax rotation Proportion of variance explained: 57,18% (total), 43,7% (1st factor). Loadings of 20 items 0,19 - 0,77. 1 item showed item complexity (loading 0,19); one item was borderline complex (loading 0,39). 4-dimensional structure could not be confirmed. | Good | **Expected correlations:** Significant positive correlation between CIVIQ scores and CEAP classification. No hypothesis and expected correlation stated on correlation between CIVIQ scores and MCS and PCS of SF-36 | CEAP ANOVA = p < 0.001 PCS SF-36: Spearman's ρ = -0,64 MCS SF-36: Spearman's ρ = -0,42 | Fair | | All items translated into Dutch language using multiple forward and backward translations. | Good |
| ***The Short Form 36 (SF-36) Health Survey*** | | | | | | | | | | |
| Garratt AM, 1993^11^ | - | - | Exploratory FA: 5-factor model Eigenvalues before rotation: 12,8; 3,8; 2,1; 1,8; 1,3 | Fair | **Expected correlations:** Correlation between SF-36 scores in varicose veins and general population. Negative correlation between SF-36 scores and referral by general practitioner. Positive correlation between SF-36 scores and general practitioners' perceptions of symptom severity. | **Mean deviation for SF-36 score in varicose veins compared with general population, corrected for age, sex and socioeconomic status:**  PF -5,3, P<0,01 PA -11,1, P<0,01 BP -8,5, P<0,01 GH -1,3, NS V -4,9, P<0,01 SF 0,2, NS EWB -8,4, P<0,01 MH -3,1, P<0,05 **Mean deviation for SF-36 score in varicose veins for referred vs. not-referred patients with general population, corrected for age, sex and socioeconomic status:** PF -5,3 (P<0,01) vs. -4,2 (NS) PA -11,6 (P<0,01) vs. -8,5 (NS) BP -9,1 (P<0,01) vs. -5,6 (NS) GH -1,4 (NS) vs. -0,1 (NS) V -5,8 (P<0,01) vs. -0,4 (NS) SF 0 (NS) vs. 1,9 (NS) EWB -8,9 (P<0,01) vs. -4,9 (NS) MH -3,7 (P<0,01) vs. 0,2 (NS)  **Statistically significant (P<0,01) mean score ordered according to general practitioners' perceptions of symptom severity.** | | Fair | - | - |
| Garratt AM, 1994^12^ | - | - | - | - | - | - | | - | - | - |
| Ruta DA, 1994^13^ | - | - | - | - | - | - | | - | - | - |
| Garratt AM, 1996^14^ | - | - | - | - | **Expected correlations:** Correlations between dimensions of SF-36 and referral, general practitioner ratings of symptom severity and complications. | **Mean score SF-36 Referred (No vs. Yes)** PF 21,75 vs. 19,73, NS PA 64,49 vs. 65,60, NS BP 70,23 vs. 69,17, NS GH 70,68 vs. 69,03, NS V 60,08 vs. 55,79, NS SF 83,62 vs. 81,68, NS EWB 70,26 vs. 68,97, NS MH 75,13 vs. 71,42, NS **GP rating (none, mild, moderate, severe)** PF 100; 82,06; 77,45; 53,41 PA 100; 80,26; 61,19; 35,71, NS BP 89,81; 81,67; 65,36; 61,90, NS GH 58,56; 77,41; 69,16; 71,86, NS V 55; 68,50; 56,03; 55, NS SF 89,81; 90; 82,59; 68,25, NS EWB 100; 84,21; 71,43; 28,57 MH 72; 78,40; 72,20; 74,86, NS **Complications (No vs. Yes)** PF 79,83 vs. 73,50, NS PA 62,68 vs. 47,50, NS BP 70,85 vs. 56,67, NS GH 73,34 vs. 75,30, NS V 57,97 vs. 54, NS SF 82,93 vs. 78,89, NS EWB 76,33 vs. 63,33, NS MH 75,56 vs. 74, NS | | Fair | - | - |
| Walters SJ, 1999^15^ | - | - | - | - | **Expected correlations:** 1. Correlations within dimensions of SF-36 and between dimensions of SF-36 and others QoL instruments. 2. Correlations between dimensions of SF-36 and age, mobility, initial ulcer size, current ulcer duration and maximum ulcer duration. | 1. Pearson's correlation coefficients within dimensions of SF-36 ranging from 0,23 - 1,00. Pearson's correlation coefficients between dimensions of SF-36 and other QoL instruments: BP with SF-MPQ r ≥ 0,43. PF with FAI r = 0,72. PF with DSI r = 0,67. V with DSI r = 0,66. 2. Mean score differences between: - Patients under and over 75 years of age, highly significant for PF, GHP and MH. - Patients able and unable to walk freely and unaided significant for PF, PA, BP, V, SF. - Median initial ulcer size ≤ or > 5,61 cm² significant for PF and MH. - Median current ulcer duration ≤ or > 7 months, and median maximum ulcer duration ≤ or > 7 years, small effect sizes for all dimensions. | | Good | - | - |
| Jull A, 2010 (2)^16^ | - | - | - | - | - | - | | - | - | - |
| Franks PJ, 2003^17^ | - | - | - | - | - | - | | - | - | - |
| Franks PJ, 2006 (1)^18^ | - | - | - | - | **Expected correlations:**  Correlation between dimensions of SF-36 and Barthel, McGill and EQ-5D. Correlation between dimensions of SF-36 and age, duration of swelling, history of cancer and presence of ulceration. | **Spearman correlation coefficients within dimensions of SF-36 and between dimensions of SF-36 and Barthel, McGill and EQ-5D:**  All correlations between domains of SF-36 significant (p<0,05). Highly correlated with all questionnaires. **Effect sizes between dimensions of SF-36 and increasing age, duration of swelling, history of cancer and presence of ulceration:**  Age ranging -0,31 - 0,54 (88% NS) Duration of swelling ranging -0,19 - 0,20 (100% NS) History of cancer ranging -0,14 - 0,40 (100% NS) Presence of ulceration ranging -0,10 - 0,24 (100% NS) | | Poor | - | - |
| Qiang cao, 2013^19^ | - | - | Exploratory FA with maximal variance rotation: 2-factor model Proportion of variance explained: 67,011% Pearson's dimension-scale coefficients: All dimensions r > 0,6 (P<0,01) | Good | - | - | | - | - | - |
| ***The Euro Quality of Life - 5 Domain (EQ-5D)*** | | | | | | | | | | |
| Iglesias CP, 2005^20^ | - | - | - | - | **Expected correlations:** Correlations between EQ-5D scores and age, degree of mobility, initial ulcer size and duration of current ulcer. | **ES (difference in means in 2 groups / full sample SD at baseline) between** - Patients ≤ and > 71 years of age: 0,1, P < 0,05 - Patients fully and partially mobile: -0,7, P < 0,05 - Median initial ulcer size ≤ and > 4 cm²: 0,0 (NS) - Mean duration of current ulcer ≤ and > 3 months: 0,0 (NS) | Fair | | - | - |
| Jull A, 2010 (1)^16^ | - | - | - | - | - | - | - | | - | - |
| Franks PJ, 2006 (2)^18^ | - | - | - | - | **Expected correlations:**  Correlation between EQ-5D scores and dimensions of SF-36, Barthel and McGill. Correlation between EQ-5D scores and age, duration of swelling, history of cancer and presence of ulceration. | **Spearman correlation coefficients:** EQ-5D highly correlated with all other questionnaires (p<0,01) **Effect sizes:**  Age -0,01, NS Duration of swelling 0,17, NS History of cancer -0,22, NS Presence of ulceration 0,03, NS | Poor | | - | - |
| ***Pediatric Quality of Life Inventory Neurofibromatosis Type 1 (PedsQL NF1, adults)*** | | | | | | | | | | |
| Nutakki K, 2013 (1)^21^ | 1. Initial outline based on literature review and clinicians' experience. 2. Pilot instrument modified by focus group/semi-structured interviews, cognitive interviews and experts' review. | Good | - | - | - | - | - | | - | - |
| Nutakki K, 2013 (2)^21^ | - | - | 1. Exploratory FA using promax rotation. 2. Multitrait scaling analysis: Item-internal consistency (correlations between items and hypothesized subscale corrected for overlap): All items > 0,4 except for one item on 'Worry' subscale. Scaling success for all subscales (mean, median): 73%, 71,4% (defined as percentage of item scaling successes relative to total number of item scaling tests with individual item scaling success, defined as number of times an item correlated higher with its hypothesized subscale construct rather than with another subscale by ≥ 2 standard errors) | Excellent | **Expected correlations:** Correlations between dimensions of PedsQL NF1 scores and self-reported health status. | **Comparing mean subscale scores between 'excellent to very good', 'good' and 'fair to poor' using one-way ANOVA (ES, P)** Physical functioning (0,51, P<0,0001) Emotional functioning (0,33, P=0,001) Social functioning (0,35, P<0,0001) Cognitive functioning (0,24, P=0,020) Communication (0,25, P=0,016) Worry (0,36, P<0,0001) Perceived physical appearance (0,22, P=0,044) Pain and hurt (0,63, P<0,0001) Paresthesia’s (0,53, P<0,0001) Skin irritation (0,44, P<0,0001) Sensation (0,26, P=0,015) Movement and balance (0,53, P<0,0001) Daily activities (0,45, P<0,0001) Fatigue (0,52, P<0,0001) Treatment anxiety (0,32, P=0,001) Total score (0,59, P<0,0001) | Fair | | - | - |
| ***Pediatric Quality of Life Inventory Neurofibromatosis Type 1 (PedsQL NF1, children, adolescents and young adults)*** | | | | | | | | | | |
| Nutakki K, 2017^22^ | 1. Initial outline based on literature review and clinicians' experience. 2. Semi-structured interviews and content analysis. 3. Operationalizing of content to items. 4. Cognitive interviews. 5. Pilot testing. | Excellent | - | - | - | - | - | | - | - |
| PCA: principal component analysis; PAF: principal axis factoring; QoL: quality of life; MAP: multitrait analysis; CEAP: comprehensive classification system for chronic venous disorders; ES: effect size; MCS: mental component scale; ANOVA: analysis of variance; PCS: physical component scale; FA: factor analysis; MLE: maximum likelihood extraction; VEINES-QoL: Venous Insufficiency Epidemiological and Economic Study-Quality of Life/Symptoms Questionnaire; NHP: Nottingham Health Profile; PF: physical functioning; PA: physical aspects; BP: bodily pain; GH: general health; V: vitality; SF: social functioning; EWB: emotional well-being; MH: mental health; NS: not significant; SF-MPQ: short-form McGill Pain Questionnaire; FAI: Frenchay Activities Index; DSI: derived single index. | | | | | | | | | | |

**References**

1 Balakrishnan K, Edwards TC, Perkins JA. Functional and symptom impacts of pediatric head and neck lymphatic malformations: developing a patient-derived instrument. *Otolaryngol Head Neck Surg* 2012; **147**: 925-31.

2 Kirkham EM, Edwards TC, Weaver EM *et al.* The Lymphatic Malformation Function (LMF) Instrument. *Otolaryngology--head and neck surgery : official journal of American Academy of Otolaryngology-Head and Neck Surgery* 2015; **153**: 656-62.

3 Launois R, Reboul-Marty J, Henry B. Construction and validation of a quality of life questionnaire in chronic lower limb venous insufficiency (CIVIQ). *Quality of life research : an international journal of quality of life aspects of treatment, care and rehabilitation* 1996; **5**: 539-54.

4 Jantet G. RELIEF study: first consolidated European data. Reflux assEssment and quaLity of lIfe improvement with micronized Flavonoids. *Angiology* 2000; **51**: 31-7.

5 Jantet G. Chronic venous insufficiency: worldwide results of the RELIEF study. Reflux assEssment and quaLity of lIfe improvEment with micronized Flavonoids. *Angiology* 2002; **53**: 245-56.

6 Lozano FS, Launois R. Quality of life (Spain and France): validation of the chronic venous insufficiency questionnaire (CIVIQ). *Methods and findings in experimental and clinical pharmacology* 2002; **24**: 425-9.

7 Erevnidou K, Launois R, Katsamouris A *et al.* Translation and validation of a quality of life questionnaire for chronic lower limb venous insufficiency into greek. *International angiology : a journal of the International Union of Angiology* 2004; **23**: 394-9.

8 Launois R, Mansilha A, Jantet G. International psychometric validation of the Chronic Venous Disease quality of life Questionnaire (CIVIQ-20). *European journal of vascular and endovascular surgery : the official journal of the European Society for Vascular Surgery* 2010; **40**: 783-9.

9 Ozdemir OC, Tonga E, Tekindal A *et al.* Cross-cultural adaptation, reliability and validity of the Turkish version of the Chronic Venous Disease Quality of Life Questionnaire (CIVIQ-20). *SpringerPlus* 2016; **5**: 381.

10 Biemans AA, van der Velden SK, Bruijninckx CM *et al.* Validation of the chronic venous insufficiency quality of life questionnaire in Dutch patients treated for varicose veins. *European journal of vascular and endovascular surgery : the official journal of the European Society for Vascular Surgery* 2011; **42**: 246-53.

11 Garratt AM, Ruta DA, Abdalla MI *et al.* The SF36 health survey questionnaire: an outcome measure suitable for routine use within the NHS? *BMJ (Clinical research ed.)* 1993; **306**: 1440-4.

12 Garratt AM, Ruta DA, Abdalla MI *et al.* SF 36 health survey questionnaire: II. Responsiveness to changes in health status in four common clinical conditions. *Quality in health care : QHC* 1994; **3**: 186-92.

13 Ruta DA, Abdalla MI, Garratt AM *et al.* SF 36 health survey questionnaire: I. Reliability in two patient based studies. *Quality in health care : QHC* 1994; **3**: 180-5.

14 Garratt AM, Ruta DA, Abdalla MI *et al.* Responsiveness of the SF-36 and a condition-specific measure of health for patients with varicose veins. *Quality of life research : an international journal of quality of life aspects of treatment, care and rehabilitation* 1996; **5**: 223-34.

15 Walters SJ, Morrell CJ, Dixon S. Measuring health-related quality of life in patients with venous leg ulcers. *Quality of life research : an international journal of quality of life aspects of treatment, care and rehabilitation* 1999; **8**: 327-36.

16 Jull A, Parag V, Walker N *et al.* Responsiveness of generic and disease-specific health-related quality of life instruments to venous ulcer healing. *Wound repair and regeneration : official publication of the Wound Healing Society [and] the European Tissue Repair Society* 2010; **18**: 26-30.

17 Franks PJ, McCullagh L, Moffatt CJ. Assessing quality of life in patients with chronic leg ulceration using the Medical Outcomes Short Form-36 questionnaire. *Ostomy/wound management* 2003; **49**: 26-37.

18 Franks PJ, Moffatt CJ, Doherty DC *et al.* Assessment of health-related quality of life in patients with lymphedema of the lower limb. *Wound repair and regeneration : official publication of the Wound Healing Society [and] the European Tissue Repair Society* 2006; **14**: 110-8.

19 Cao Q, Tian Y, Sai LM. [Roles of short form-36 health survey scale in the evaluation of quality of life in patients with deep vein thrombosis]. *Zhonghua yi xue za zhi* 2013; **93**: 1615-8.

20 Iglesias CP, Birks Y, Nelson EA *et al.* Quality of life of people with venous leg ulcers: a comparison of the discriminative and responsive characteristics of two generic and a disease specific instruments. *Quality of life research : an international journal of quality of life aspects of treatment, care and rehabilitation* 2005; **14**: 1705-18.

21 Nutakki K, Hingtgen CM, Monahan P *et al.* Development of the adult PedsQL neurofibromatosis type 1 module: initial feasibility, reliability and validity. *Health and quality of life outcomes* 2013; **11**: 21.

22 Nutakki K, Varni JW, Steinbrenner S *et al.* Development of the pediatric quality of life inventory neurofibromatosis type 1 module items for children, adolescents and young adults: qualitative methods. *Journal of neuro-oncology* 2017; **132**: 135-43.
